# Genome-wide identification and characterization of *DCL*, *AGO*, and *RDR* gene families and their abiotic stress responses in alfalfa (*Medicago sativa* L.)

**DOI:** 10.3389/fpls.2026.1753305

**Published:** 2026-02-16

**Authors:** Ming Xu, Hao Liu, Kai Zhu, Tian Zhang, Ruicai Long, Qingchuan Yang, Fei He, Changhong Guo

**Affiliations:** 1 College of Life Science and Technology, Harbin Normal University, Harbin, China; 2 Institute of Animal Science, Chinese Academy of Agricultural Sciences, Beijing, China

**Keywords:** abiotic stress, AGO, DCL, gene family, *Medicago sativa* L., RDR

## Abstract

Dicer-like (DCL), Argonaute (AGO), and RNA-dependent RNA polymerase (RDR) proteins are core components of the plant RNA silencing pathway and play critical roles in plant growth, development, and stress adaptation. In this study, we performed the first genome-wide analysis of these gene families in autotetraploid alfalfa (*Medicago sativa* L.), identifying 31 *MsDCL*, 82 *MsAGO*, and 52 *MsRDR* genes, followed by systematic characterization. Phylogenetic analysis classified these genes into distinct evolutionary clades, and gene duplication events—particularly segmental duplications—were identified as the primary drivers of family expansion. Promoter analysis revealed an abundance of hormone- and stress-responsive *cis*-elements in their regulatory regions. Expression profiling across six tissues indicated both constitutive and tissue-specific expression patterns. Transcriptome sequencing under salt and drought stress identified six core stress-responsive candidate genes—*MsDCL11*, *MsAGO54*, *MsAGO60*, *MsAGO73*, *MsAGO76*, and *MsRDR37*—which were further validated by RT-qPCR. These findings provide a theoretical foundation for future functional studies of RNA silencing pathway genes and offer valuable genetic resources for the molecular breeding of stress-resilient alfalfa.

## 1 Introduction

RNA silencing is a sequence-specific gene regulation process mediated by small RNAs (sRNAs), which typically consist of 21 to 24 nucleotides (nt), including the small interfering RNA (siRNA) and microRNA (miRNA) classes (Fang and Qi, 2016). RNA silencing plays a crucial role in plant growth, development, and responses to biotic and abiotic stresses (Lu et al., 2008; Chen, 2009; Yu and Wang, 2020). The establishment of RNA silencing pathways depends on three core protein families: Dicer-like (DCL), Argonaute (AGO), and RNA-dependent RNA polymerase (RDR), which participate in various physiological and defense processes in plants (Baulcombe, 2004). RNA silencing mechanisms can be divided into three stages: initiation, maintenance, and signal amplification (Baulcombe, 2004). First, RDR catalyzes the synthesis of double-stranded RNA (dsRNA) from single-stranded RNA (ssRNA). Then, DCL processes dsRNA into 21 to 24 nt sRNAs (Carmell and Hannon, 2004). These sRNAs are subsequently incorporated into RNA-induced silencing complexes (RISCs), which contain AGO proteins. Within RISC, AGO mediates mRNA cleavage, translational repression, or heterochromatin formation (Bologna and Voinnet, 2014). During signal amplification, RDR utilizes primary sRNAs as primers to synthesize new dsRNAs, which are then processed into secondary sRNAs, thereby reinforcing the silencing signal (Sijen et al., 2001).

DCL enzymes belong to the RNase III family and specifically cleave dsRNA into sRNAs ranging from 21 to 24 nt (Carmell and Hannon, 2004). In *Arabidopsis thaliana*, four *DCL* genes (*DCL1* to *DCL4*) have been identified (Voinnet, 2009). DCL1 primarily processes microRNA precursors into mature miRNAs (Hiraguri et al., 2005). DCL2 is responsible for generating both viral-derived and endogenous 22 nt siRNAs (Ding and Voinnet, 2007; Taochy et al., 2017; Wang et al., 2018). DCL3 produces 24 nt heterochromatic siRNAs (hc-siRNAs) from Pol IV transcripts (Blevins et al., 2015; Wang et al., 2021), while DCL4 is essential for the production of 21 nt phased secondary siRNAs (phasiRNAs), including trans-acting siRNAs (tasiRNAs) and reproductive phasiRNAs (Liu et al., 2020).

AGO proteins are typically large and contain several conserved domains, including the N-terminal, PAZ, MID, and PIWI domains. The MID domain, together with the PIWI domain, forms a positively charged pocket that is critical for sRNA loading (Jinek and Doudna, 2009; Meister, 2013; Liang et al., 2023). The PIWI domain exhibits structural and functional similarity to RNase H and is responsible for the “Slicer” activity of some AGO proteins (Yan et al., 2003). In *A. thaliana*, ten *AGO* genes have been identified, each contributing to diverse sRNA-mediated processes (Vaucheret, 2008). Phylogenetically, AGO proteins are classified into three major clades: AGO1/5/10, AGO4/6/8/9, and AGO2/3/7 (Zhang et al., 2015). Among these, AGO1 is the key effector of miRNA- and siRNA-mediated mRNA degradation and translational repression (Baumberger and Baulcombe, 2005; Qi et al., 2005; Brodersen et al., 2008), while AGO4 and AGO6 can participate in the RNA silencing mediated by 24 nt siRNAs (Xie et al., 2004; Zheng et al., 2007; Mi et al., 2008).

Members of the *RDR* gene family possess a conserved RNA-dependent RNA polymerase (RdRP) domain, which is essential for the initiation and amplification of silencing signals (Schiebel et al., 1998). RDR catalyzes the conversion of ssRNA into dsRNA, which is subsequently processed by DCL to initiate a new round of RNA silencing (Wassenegger and Krczal, 2006). The *A. thaliana* genome encodes six *RDR* genes (Bologna and Voinnet, 2014). Among them, RDR1 is primarily involved in the biogenesis of virus-derived siRNAs (Cao et al., 2014), RDR2 is critical for the production of hc-siRNAs from Pol IV transcripts (Chan et al., 2004). and RDR6 contributes to the generation of phasiRNAs (Kumakura et al., 2009). However, the biological role functions and substrates of RDR3, RDR4, and RDR5 remain largely unknown (Wassenegger and Krczal, 2006).

*DCL*, *AGO*, and *RDR* gene families have been identified in various plant species, and some members have been functionally characterized. For example, 32 genes (eight *OsDCLs*, 19 *OsAGOs*, five *OsRDRs*) were identified in rice (*Oryza sativa* L.) (Kapoor et al., 2008), 28 genes (five *ZmDCLs*, 18 *ZmAGOs*, five *ZmRDRs*) in maize (*Zea mays* L.) (Qian et al., 2011), 144 genes (31 *TaDCLs*, 82 *TaAGOs*, 31 *TaRDRs*) in wheat (*Triticum aestivum* L.) (Mishra et al., 2023), and 35 genes (seven *GmDCLs*, 21 *GmAGOs*, seven *GmRDRs*) in soybean (*Glycine max* L.) (Liu et al., 2014). These studies on the functional mechanisms of members of *DCL*, *AGO*, and *RDR* gene families reveal that *DCL*, *AGO*, and *RDR* genes not only play essential roles in plant development but also contribute significantly to abiotic stress responses. For instance, in pea (*Pisum sativum*), AGO1 interacts with Psp68, a DEAD-box protein, to enhance salt tolerance in rice (Banu et al., 2015). Moreover, abiotic stresses such as drought and salinity induce differential expression of *DCL*, *AGO*, and *RDR* genes in species like rice (Kapoor et al., 2008), cucumber (*Cucumis sativus*) (Gan et al., 2017), and tea plants (*Camellia sinensis*) (Krishnatreya et al., 2021).

Alfalfa (*Medicago sativa* L.), known as the “Queen of Forages,” is a widely cultivated legume valued for its high biomass yield and superior nutritional quality (Nasrollahi et al., 2023; Zhao et al., 2023). Alfalfa is an autotetraploid and cross-pollinating species with a highly heterozygous genome, which poses unique challenges for genome analysis and gene family identification. In this study, we utilized a phased genome assembly of the cultivar ‘Xinjiang Daye’ that resolves four haplotypes across 32 chromosomes, enabling accurate detection and discrimination of homeologous genes. Previous studies have suggested that *DCL*, *AGO*, and *RDR* genes may participate in alfalfa’s abiotic stress responses. For example, Lundell et al. reported that *Rhizobium* inoculation enhances miRNA-mediated post-transcriptional regulation in salt-tolerant alfalfa by upregulating *AGO* expression and cooperating with epigenetic mechanisms to improve salt stress tolerance (Lundell and Biligetu, 2024). However, a systematic genome-wide analysis of *DCL*, *AGO*, and *RDR* gene families in alfalfa remains lacking. In this study, we conducted a comprehensive genome-wide identification and characterization of the *DCL*, *AGO*, and *RDR* gene families in alfalfa. Our analyses included phylogenetic reconstruction, gene structure analysis, conserved motif identification, chromosomal localization, and collinearity evaluation. Furthermore, we analyzed RNA-seq data to investigate the expression patterns of these genes across different tissues and under various abiotic stress conditions. To confirm their stress-associated expression patterns, the differential expression of the selected genes under salt and drought stress was quantified using RT-qPCR. Together, this work provides important insights into the functional roles of *DCL*, *AGO*, and *RDR* genes in alfalfa, laying a foundation for their future applications in molecular breeding for stress resilience.

## 2 Materials and methods

### 2.1 Identification of putative *DCL*, *AGO*, and *RDR* genes in alfalfa genome

The alfalfa genome used in this study was obtained from the Alfalfa Genome Project (https://figshare.com/projects/whole_genome_sequencing_and_assembly_of_Medicago_sativa/66380) (Chen et al., 2020b). This assembly represents a phased genome comprising four haplotypes across 32 chromosomes, which facilitates the resolution of duplicated and homeologous loci in autotetraploid alfalfa. The genome sequence of *Arabidopsis thaliana* was retrieved from the TAIR database (https://www.arabidopsis.org/), while the genomes of other plant species, including *Medicago truncatula*, *Glycine max*, and *Oryza sativa*, were downloaded from Ensembl Plants (https://plants.ensembl.org/index.html). The *DCL*, *AGO*, and *RDR* gene family members in *A. thaliana* were identified based on previously published studies (**Supplementary Table S1**) (Podder et al., 2023). To identify putative *DCL*, *AGO*, and *RDR* genes in the alfalfa genome, we performed BLASTP searches using known *A. thaliana* protein sequences as queries, with an E-value threshold of ≤ 1×10^−10^. The resulting candidate genes were further validated using the Conserved Domain Database (CDD, https://www.ncbi.nlm.nih.gov/cdd/) to confirm the presence of characteristic domains specific to each gene family: DEAD, Helicase-C, Dicer-dimer, PAZ, and DSRM domains for DCL proteins; ArgoN, PAZ, MID, and PIWI domains for AGO proteins; and RdRP domain for RDR proteins. Any sequences lacking these essential conserved domains were excluded from further analysis.

### 2.2 Bioinformatics analysis of MsDCL, MsAGO, and MsRDR proteins

Using TBtools v2.156 (Chen et al., 2020a), we extracted information on protein sequence length, molecular weight, and theoretical isoelectric point (pI) for the identified MsDCL, MsAGO, and MsRDR proteins from the GFF annotation file of the alfalfa reference genome. The subcellular localization of these proteins was predicted using the online tool WoLF PSORT (https://wolfpsort.hgc.jp/).

### 2.3 Phylogenetic analysis, gene structure, and conserved motif identification

Multiple sequence alignments of DCL, AGO, and RDR protein sequences from *Medicago sativa*, *A. thaliana*, *Glycine max*, and *Oryza sativa* were performed using MEGA11. The gene IDs for *DCL*, *AGO*, and *RDR* family members in *G. max* and *O. sativa* were obtained from previously published studies (Kapoor et al., 2008; Liu et al., 2014). A phylogenetic tree was constructed using the neighbor-joining (NJ) method with 1,000 bootstrap replicates to assess tree reliability. The resulting tree was visualized using the online tool Evolgenius (https://evolgenius.info//evolview-v2/#login). To identify conserved motifs in MsDCL, MsAGO, and MsRDR proteins, the MEME Suite (https://meme-suite.org/meme/index.html) was used with default parameters, except that the maximum number of motifs was set to 10. Gene structure information for *MsDCL*, *MsAGO*, and *MsRDR* was extracted from the GFF annotation file of the alfalfa genome. Visualization of the results was performed using TBtools.

### 2.4 Chromosomal localization, gene duplication events and collinearity analysis

To investigate the collinearity relationships of *DCL*, *AGO*, and *RDR* genes both within alfalfa and across different species, the MCScanX tool was employed (Wang et al., 2012). Chromosomal localization of *MsDCL*, *MsAGO*, and *MsRDR* family members was visualized using TBtools. Tandem duplication events were defined as two or more genes with sequence similarity exceeding 70% located within a 200 kb region on the same chromosome, a commonly used operational criterion in plant gene-family studies for capturing tandem arrays that may span larger intergenic regions. Pairwise sequence similarity between genes was assessed using MEGA11. To identify tandemly duplicated *MsDCL*, *MsAGO*, and *MsRDR* genes, their chromosomal positions were analyzed and compared accordingly.

### 2.5 Analysis of *cis*-acting element in promoter regions

TBtools was used to extract the 2,000 bp upstream promoter regions of the *MsDCL*, *MsAGO*, and *MsRDR* genes. *Cis*-acting regulatory elements within these sequences were identified using the PlantCARE online database (http://bioinformatics.psb.ugent.be/webtools/plantcare/html/). After identification, the distribution and functional classification of these elements were visualized using TBtools.

### 2.6 Expression pattern analysis using RNA-seq data

RNA-seq datasets from six alfalfa tissues (roots, elongated stems, pre-elongated stems, leaves, flowers, and nodules; project ID: SRP055547) and from plants subjected to abiotic stress (salt and drought treatments; run accessions: SRR7160313–SRR7160357) were obtained from the NCBI Sequence Read Archive (SRA) (O’Rourke et al., 2015; Dong et al., 2021). Cleaned reads were aligned to the Xinjiang Daye reference genome using TopHat2. Gene expression levels were quantified using FPKM (Fragments Per Kilobase of transcript per Million mapped reads). Differential expression analysis under stress conditions was performed with DESeq2, applying thresholds of *p*adj < 0.05 and |log_2_FC| ≥ 1 to identify significant changes. The expression profiles of *MsDCL*, *MsAGO*, and *MsRDR* family members were subsequently analyzed and visualized using TBtools.

### 2.7 Plant materials, growth and stress conditions, and RT-qPCR analysis

Due to limited material availability, the alfalfa cultivar Zhongmu No. 4 (*Medicago sativa* L.) was used for RT-qPCR assays in this study, and seeds of this cultivar were provided by the Institute of Animal Science, Chinese Academy of Agricultural Sciences. Prior to cultivation, the seeds were stratified at 4 °C for three days. Seedlings were then grown in a greenhouse under controlled conditions for two weeks, with a 16 h light/8 h dark photoperiod, 70–80% relative humidity, and day/night temperatures of 24 °C/20 °C. Stress treatment was conducted using a hydroponic method. Two-week-old seedlings were transferred to containers containing a 1/2 strength Hoagland nutrient solution for a 24-hour acclimation period. Subsequently, the nutrient solution was completely replaced with a 1/2 strength Hoagland solution containing 15% PEG 6000 (for drought stress) or 200 mM NaCl (for salt stress) to initiate the stress treatment. The control group continued to be maintained in a standard 1/2 strength Hoagland solution. The volume of nutrient solution in each container was kept consistent across all containers and was not replenished throughout the entire 24-hour treatment period to maintain a constant stress intensity. For both treatments, leaf samples were collected at 0, 6, 12, and 24 hours post-treatment, with the 0-hour samples serving as controls. Each treatment included three biological replicates, with five seedlings pooled per replicate. Untreated control plants were grown under the same greenhouse conditions. Total RNA was extracted from all samples using TRIzol reagent (Invitrogen, USA), following the manufacturer’s instructions. First-strand cDNA was synthesized using the EasyScript First-Strand cDNA Synthesis Kit (TransGen Biotech, China). Gene-specific primers were designed using Primer 5.0 (**Supplementary Table S2**). RT-qPCR was performed using SYBR Premix Ex Taq (Takara, Japan) on a 7500 Real-Time PCR System (Applied Biosystems, Foster City, CA, USA). Each sample was analyzed in technical triplicate, and gene expression levels were normalized against the alfalfa *ACTIN* gene. The expression stability of the A*CTIN* gene under salt and drought stress conditions was verified in our preliminary experiments, a finding that aligns with previous studies in alfalfa where *ACTIN* has been confirmed as a reliable internal reference gene under comparable abiotic stress treatments (Li et al., 2014). Therefore, it was selected as the internal control for normalization of RT−qPCR data in this study. Relative expression was calculated using the 2^−ΔΔCT^ method.

## 3 Results

### 3.1 Genome-wide identification of *AGO*, *DCL* and *RDR* genes in alfalfa

Based on conserved domain structures and previously characterized RNA silencing pathway genes in *Arabidopsis thaliana*, a BLAST search was performed against the genome of *Medicago sativa* cv. Xinjiang Daye. A total of 31 *MsDCL*, 82 *MsAGO*, and 52 *MsRDR* genes were identified in the alfalfa genome. These genes were designated as *MsDCL1* to *MsDCL31*, *MsAGO1* to *MsAGO82*, and *MsRDR1* to *MsRDR52*, respectively, according to their chromosomal locations. The detailed features of these genes and their encoded proteins are listed in **Supplementary Tables S3** and **S4**.

Within the *MsDCL* gene family, *MsDCL6* encodes the shortest protein (1,140 amino acids, 129.52 kDa), whereas *MsDCL20* encodes the longest (1,889 amino acids, 212.23 kDa). The theoretical isoelectric point (pI) of MsDCL proteins ranges from 5.87 (MsDCL19) to 7.99 (MsDCL24), and their instability indices range from 40.00 (MsDCL6) to 45.48 (MsDCL11). For the *MsAGO* gene family, *MsAGO73* encodes the smallest protein (636 amino acids, 71.92 kDa), while *MsAGO66* encodes the largest (1,129 amino acids, 124.64 kDa). The pI values of MsAGO proteins range from 8.43 (MsAGO82) to 9.77 (MsAGO59), and the instability index ranges from 37.70 (MsAGO31) to 51.83 (MsAGO54). Within the *MsRDR* family, *MsRDR52* encodes the shortest protein (794 amino acids, 90.52 kDa), while *MsRDR14* encodes the longest (1,396 amino acids, 159.48 kDa). The pI values of MsRDR proteins range from 6.21 (MsRDR29) to 9.11 (MsRDR12), and their instability indices range from 36.05 (MsRDR44) to 47.98 (MsRDR47). Comparative analysis revealed that MsDCL proteins are generally longer and heavier than MsAGO and MsRDR proteins, whereas MsAGO proteins tend to exhibit higher isoelectric points. Subcellular localization prediction (**Supplementary Table S4**) showed that most MsDCL proteins are targeted to the chloroplast (23) or nucleus (8). Among the MsAGO proteins, 66 are predicted to localize in the nucleus, with others distributed across the chloroplast (8), mitochondrion (5), cytosol (2), and peroxisome (1). Most MsRDR proteins are also predicted to be nuclear-localized (46), with a few targeted to the cytosol (4), chloroplast (1), or mitochondrion (1).

**Figure 1:**
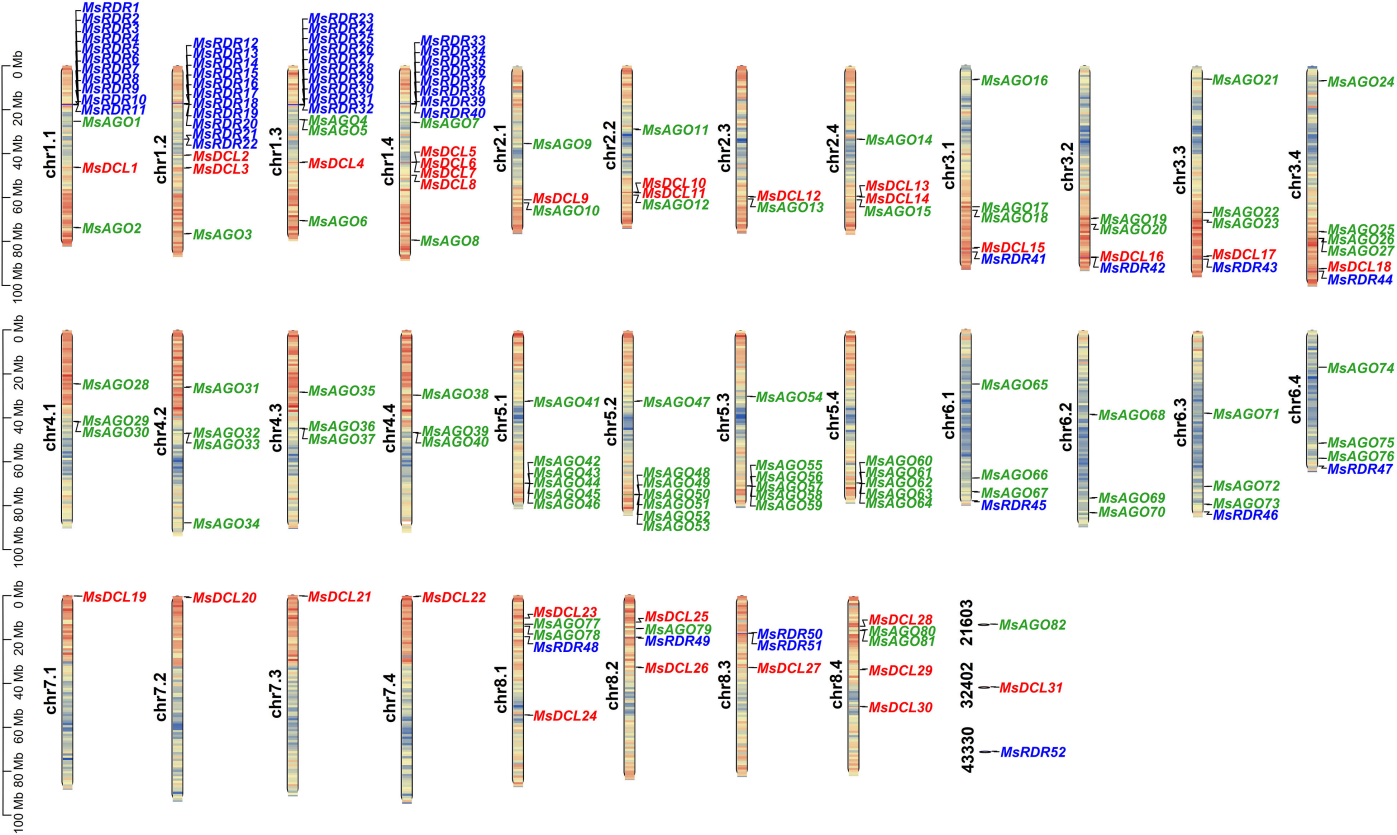
Chromosomal distribution of *MsDCL*, *MsAGO*, and *MsRDR* genes in the alfalfa genome. Chromosome coloration indicates gene density, with a gradient from blue (low density) to red (high density). The scale on the left represents chromosome length in megabase pairs (Mb). Genes belonging to the *DCL*, *AGO*, and *RDR* families are labeled in red, green, and blue, respectively.

All identified RNA silencing pathway genes are distributed across the 32 assembled chromosomes of the alfalfa genome, with three genes located on unanchored scaffolds (21603, 32402, and 43330). Specifically, 30 MsDCL genes (*MsDCL1*–*MsDCL30*) are distributed across 20 chromosomes, with none detected on chromosomes chr4.1–4.4, chr5.1–5.4, or chr6.1–6.4. *MsDCL31* is located on unanchored scaffold 32402. Similarly, 81 *MsAGO* genes (*MsAGO1*–*MsAGO81*) are located on 27 chromosomes, while *MsAGO82* is present on scaffold 21603, with no *MsAGO* genes found on chr7.1–7.4 or chr8.3. The *MsRDR* family consists of 51 genes (*MsRDR1*–*MsRDR51*) spread across 14 chromosomes, and *MsRDR52* is located on scaffold 43330. Notably, *MsRDR* genes are absent from chr2.1–2.4, chr4.1–4.4, chr5.1–5.5, chr6.2, chr7.1–7.4, and chr8.4 (**Figure 1**).

### 3.2 Phylogenetic analysis of *DCL*, *AGO* and *RDR* genes families

**Figure 2:**
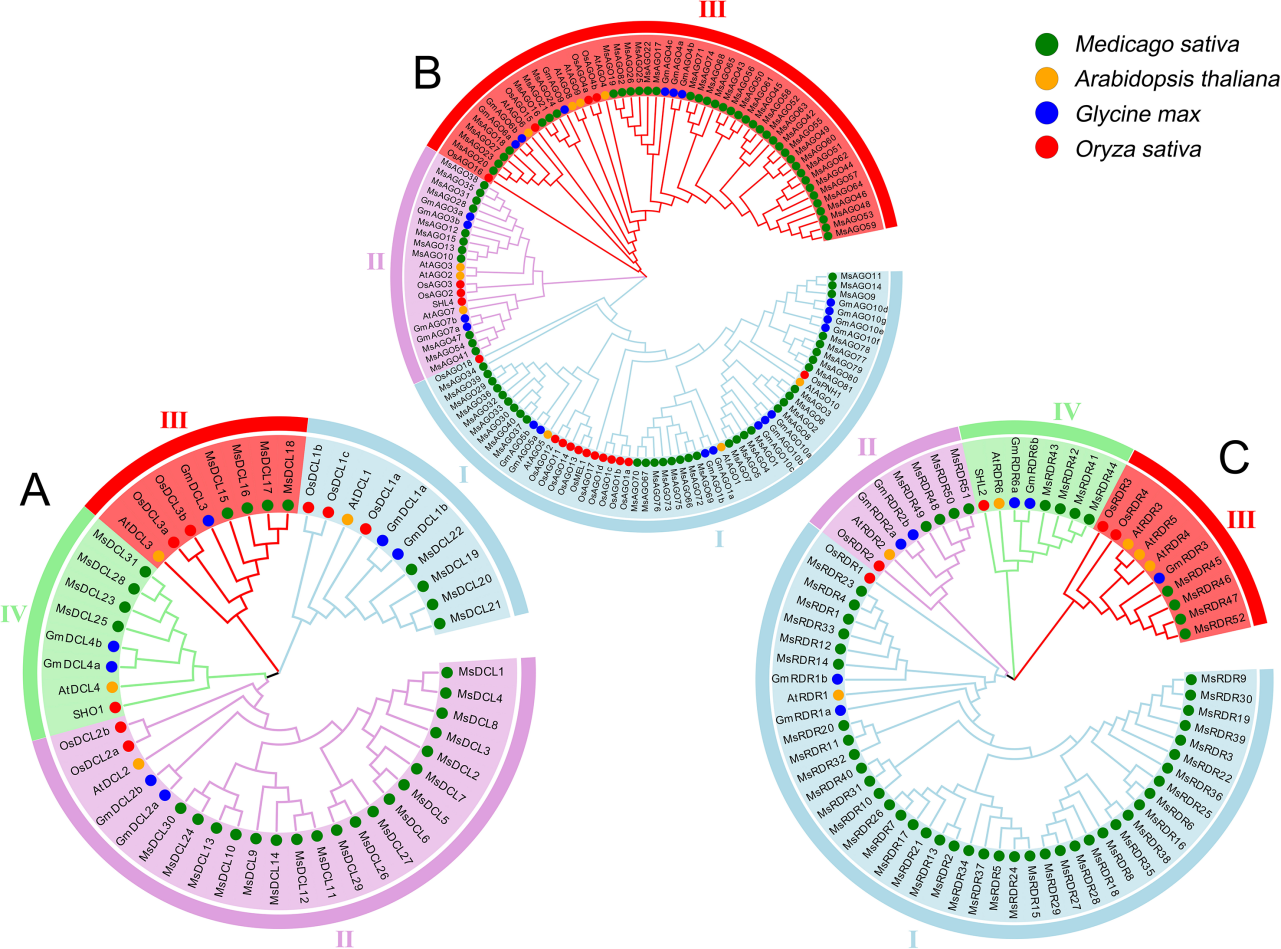
**(A)** Phylogenetic tree of the *DCL* gene family. **(B)** Phylogenetic tree of the *AGO* gene family. **(C)** Phylogenetic tree of the *RDR* gene family.

Phylogenetic trees of the *DCL*, *AGO*, and *RDR* gene families were constructed based on protein sequences from *M. sativa*, *A. thaliana*, *G. max*, and *O. sativa* (**Figure 2**). The *DCL* genes were grouped into four distinct clades (I–IV) according to their evolutionary relationships. Each of the four *A. thaliana DCL* genes was assigned to a separate clade. Among the *M. sativa* genes, clades I–IV contained 4, 19, 4, and 4 *MsDCL* genes, respectively. Similarly, the *AGO* genes were divided into three major clades (I–III). Consistent with previous classifications (Vaucheret, 2008; Zhang et al., 2015), *AtAGO1/5/10* were grouped into clade I, *AtAGO2/3/7* into clade II, and *AtAGO4/6/8/9* into clade III. The *M. sativa* genes were distributed as follows: 33 *MsAGO* genes in clade I, 11 in clade II, and 38 in clade III. The *RDR* gene family was classified into four clades (I–IV). Clade III included the three *A. thaliana* genes *AtRDR3*, *AtRDR4*, and *AtRDR5*, while clades I, II, and IV each contained a single *A. thaliana RDR* gene. In *M. sativa*, clades I–IV contained 40, 4, 4, and 4 *MsRDR* genes, respectively.

### 3.3 Gene structure and conserved motif analysis

To investigate the structural characteristics of RNA silencing pathway genes in alfalfa, conserved motifs were identified using the MEME online tool. A total of 10 conserved motifs were detected for each gene family, as shown in **Supplementary Figure S1**. The types and numbers of motifs varied across individual gene members. For the MsDCL proteins (**Supplementary Figure S2**), most members contained all 10 motifs. Notably, MsDCL6 lacked motif 2; MsDCL15–23, MsDCL25, and MsDCL28 lacked motif 10; MsDCL31 lacked motifs 3, 6, 7, 8, and 10. All other MsDCL proteins contained the full set of motifs. Regarding MsAGO proteins (**Supplementary Figure S3**), 76 proteins contained all 10 motifs. However, MsAGO48 and MsAGO82 lacked motif 2; MsAGO73 lacked motif 5; MsAGO21 lacked motifs 1 and 2; MsAGO59 lacked motifs 1, 2, 4, and 8; and MsAGO19 retained only motifs 5, 6, 7, and 10. Among the MsRDR proteins (**Supplementary Figure S2**), 40 members possessed all 10 motifs. MsRDR1, MsRDR4, MsRDR23, MsRDR33, MsRDR48, and MsRDR51 lacked motif 8. MsRDR46, MsRDR47, and MsRDR52 were missing motifs 3, 4, 6, 7, and 9, while MsRDR45 lacked motifs 2, 3, 4, 6, 7, and 9.

In addition to conserved motifs, protein domain architectures were analyzed. All MsDCL proteins possessed five core domains: Ribonuclease_3, Helicase_C, Dicer_dimer, PAZ, and DSRM. However, the DEAD domain was absent in MsDCL19–22, which instead contained the RESIII domain, a feature unique to these proteins (**Supplementary Figure S2**). All MsAGO proteins contained the Piwi domain (Piwi-like in MsAGO19 and MsAGO59), PAZ, ArgoL1, and ArgoL2 domains. The ArgoN domain was missing in MsAGO34 and MsAGO73. The Rib_recp_KP_reg domain was exclusively found in MsAGO33 and MsAGO37, while the Gly-rich_Ago1 domain was unique to MsAGO66, MsAGO67, MsAGO69, MsAGO70, MsAGO72, MsAGO75, and MsAGO76. The Herpes_TAF50 domain was present only in MsAGO31 (**Supplementary Figure S3**). All MsRDR proteins contained the canonical RdRP domain. Notably, MsRDR14 harbored two unique domains, HLH and ZapB, while MsRDR48–51 were characterized by the exclusive presence of the RRM_SF domain (**Supplementary Figure S2**). Therefore, the presence of family-specific or member-specific domains suggests that *MsDCL*, *MsAGO*, and *MsRDR* genes may have functional specialization or diversification within the RNA silencing pathways.

### 3.4 Gene duplication events and collinearity analysis of *MsDCL*, *MsAGO* and *MsRDR* genes in alfalfa

Gene duplication plays a pivotal role in the evolution of gene families by facilitating the emergence of novel genes and functions. Both segmental and tandem duplications are key mechanisms driving gene family expansion. In this study, a total of 20 tandem duplication events were identified among the *MsDCL*, *MsAGO*, and *MsRDR* gene families. For example, tandem duplications were observed between *MsRDR2*–*MsRDR11* on chr1.1, with sequence similarities ranging from 80.6% to 98.5%, and between *MsAGO4* and *MsAGO5* on chr1.3, with 100% sequence similarity. Among these tandem duplication events, 3 involved *MsDCL* genes, 9 involved *MsAGO* genes, and 8 involved *MsRDR* genes. Notably, seven tandem duplication events of *MsRDR* genes were detected across chr1.1, chr1.2, chr1.3, and chr1.4, with events 2, 4, 6, and 8 each encompassing more than seven *MsRDR* genes (**Figure 1**; **Supplementary Table S5**). Further details on these tandem duplication events are provided in **Supplementary Table S5**.

**Figure 3:**
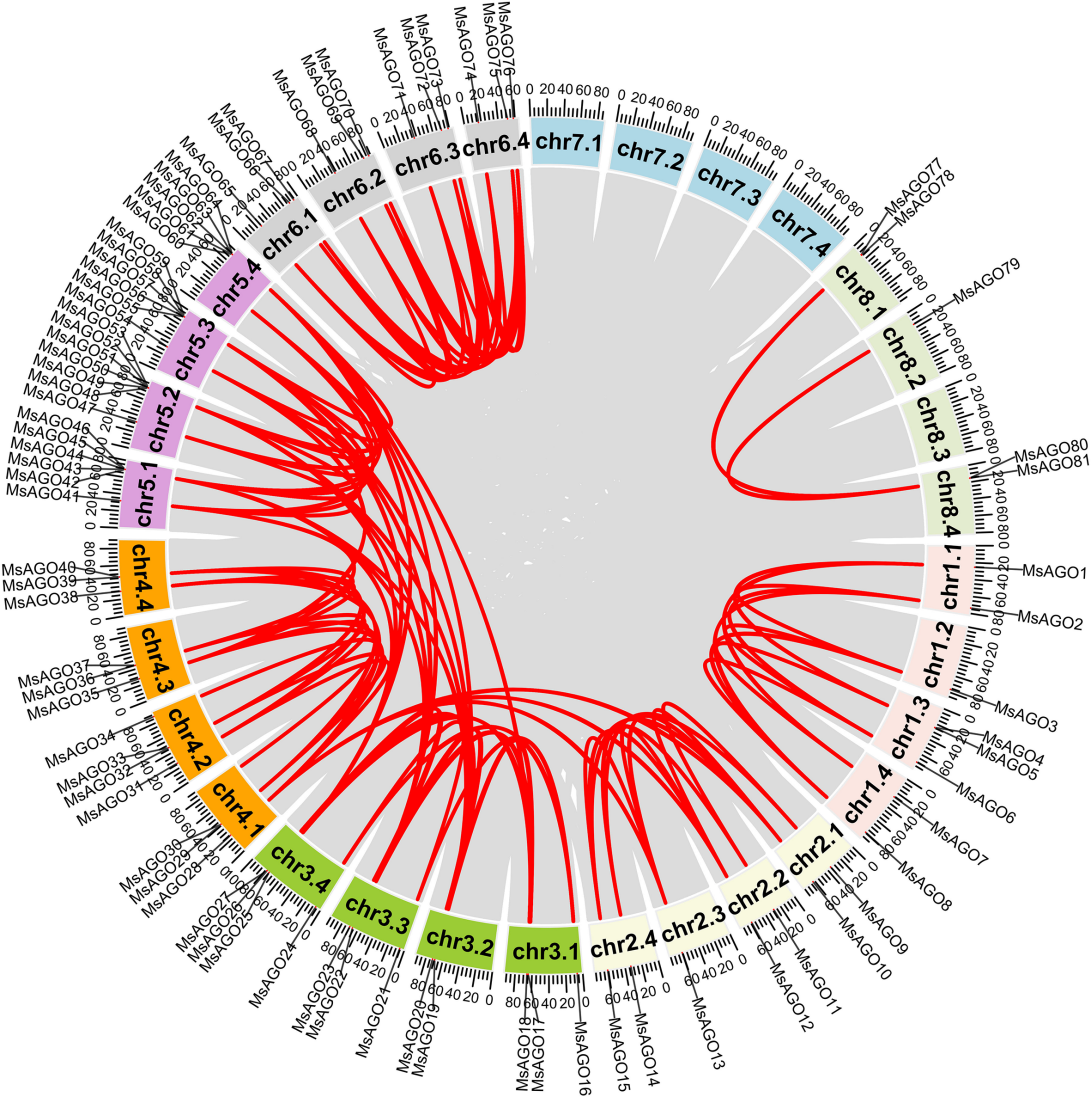
Syntenic relationships of *MsAGO* genes in alfalfa. The outermost colored blocks represent the 32 alfalfa chromosomes, with the chromosomal positions of *MsAGO* genes indicated along the circle. Gray ribbons denote syntenic blocks, while red lines highlight segmental duplication events within the genome.

In addition to tandem duplications, a total of 164 segmental duplication events were identified across the alfalfa genome (**Supplementary Table S6**). Among these, 28 were associated with *MsDCL* genes (**Supplementary Figure S4A**), 99 with *MsAGO* genes (**Figure 3**), and 37 with *MsRDR* genes (**Supplementary Figure S4B**). All segmental duplications involving *MsDCL* and *MsRDR* genes occurred between homologous chromosomes. For instance, segmental duplication was detected between *MsDCL2* on chr1.2 and *MsDCL5* on chr1.4, as well as between *MsRDR1* on chr1.1 and *MsRDR12* on chr1.2 (**Supplementary Figure S4**). In contrast, *MsAGO* genes exhibited segmental duplications on both homologous and non-homologous chromosomes. For example, a duplication event was identified between *MsAGO10* on chr2.1 and *MsAGO28* on chr4.1 (**Figure 3**).

### 3.5 Collinearity analysis of *MsDCL*, *MsAGO*, and *MsRDR* genes across species

**Figure 4:**
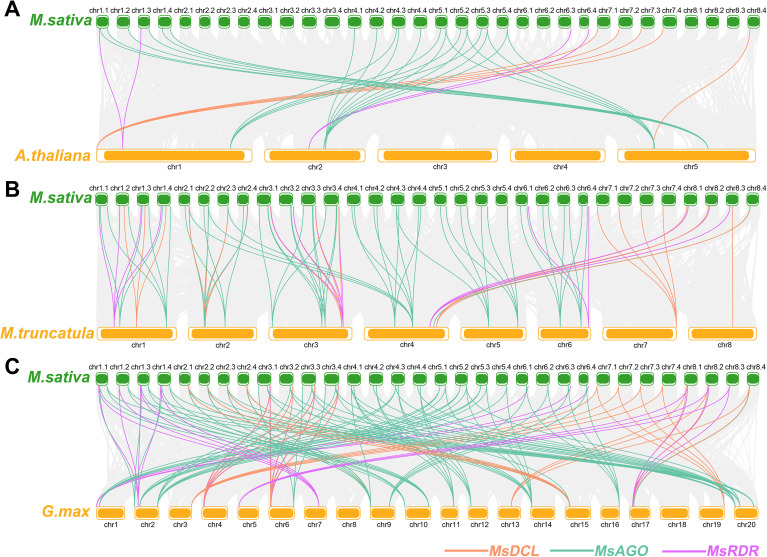
**(A)** Collinearity analysis of *MsDCL, MsAGO* and *MsRDR* genes between *M. sativa* and *A. thaliana*. **(B)** Collinearity analysis of *MsDCL, MsAGO* and *MsRDR* genes between *M. sativa* and *M. truncatula*. **(C)** Collinearity analysis of *MsDCL, MsAGO* and *MsRDR* genes between *M. sativa* and *G. max*.

To further elucidate the evolutionary mechanisms of the *DCL*, *AGO*, and *RDR* gene families, we performed a comparative collinearity analysis between *M. sativa* and three representative plant species: *A. thaliana*, *M. truncatula*, and *G. max* (**Figure 4**). Between *M. sativa* and *A. thaliana*, a total of 31 segmentally duplicated gene pairs were identified, including 5 involving *MsDCL* genes, 22 involving *MsAGO* genes, and 4 involving *MsRDR* genes. In the comparison with *M. truncatula*, 85 segmental duplication events were detected, comprising 19 *MsDCL*-related pairs, 53 *MsAGO*-related pairs, and 13 *MsRDR*-related pairs. Likewise, synteny analysis with *G. max* revealed 132 segmentally duplicated gene pairs, including 30 related to *MsDCL* genes, 79 to *MsAGO* genes, and 23 to *MsRDR* genes.

### 3.6 *Cis*-acting element analysis of promoter regions

**Figure 5:**
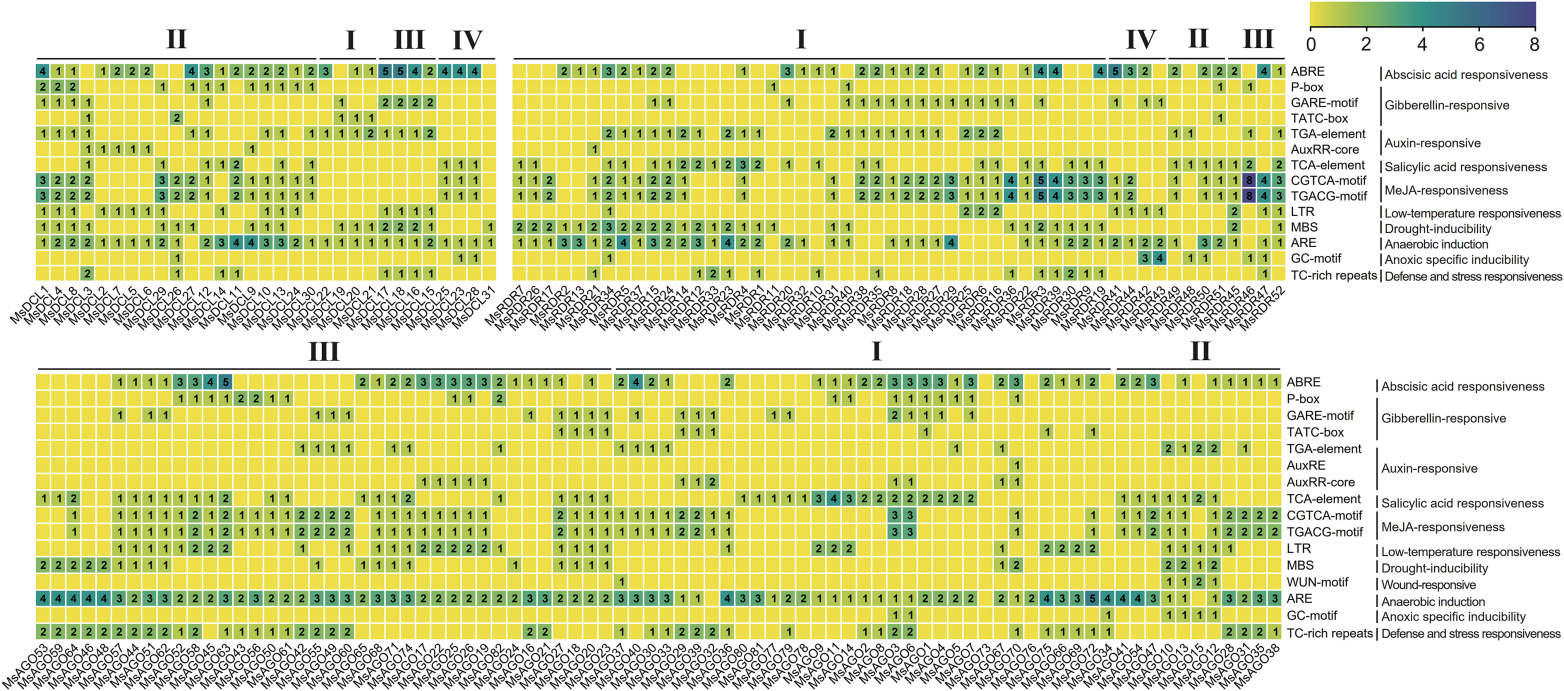
Distribution of hormone- and stress-responsive *cis*-elements in the promoter regions of *MsDCL*, *MsAGO*, and *MsRDR* genes. The color gradient indicates the abundance of each element, ranging from low (yellow) to high (blue). Roman numerals represent different gene subgroups. Right panel annotations list the *cis*-elements and their associated biological functions.

*Cis*-acting regulatory elements in the promoter regions of *MsDCL*, *MsAGO*, and *MsRDR* genes were identified using the PlantCARE database. These elements were classified into three major categories: plant growth and development, hormonal and stress responses, and light responsiveness (**Supplementary Table S7**). Sixteen hormone- and stress-responsive cis-elements were selected for further analysis (**Figure 5**). Among these, the *MsAGO* family contained all 16 types, while the *MsDCL* and *MsRDR* families collectively harbored 14 of the 16 types (absent: AuxRE and WUN-motif). In the *MsDCL* family, ABRE was the most frequently identified *cis*-element, suggesting its potential involvement in the abscisic acid signaling pathway. In contrast, ARE was the most abundant in the promoters of *MsAGO* genes, whereas the promoters of *MsRDR* genes were enriched for the CGTCA and TGACG motifs, implying their potential responsiveness to methyl jasmonate signaling. These results, based on bioinformatics predictions, indicate that *MsDCL*, *MsAGO*, and *MsRDR* genes may play distinct roles in hormone-mediated stress responses, though their specific functions require further experimental validation.

### 3.7 Expression profiling of RNA silencing pathway genes across alfalfa tissues

**Figure 6:**
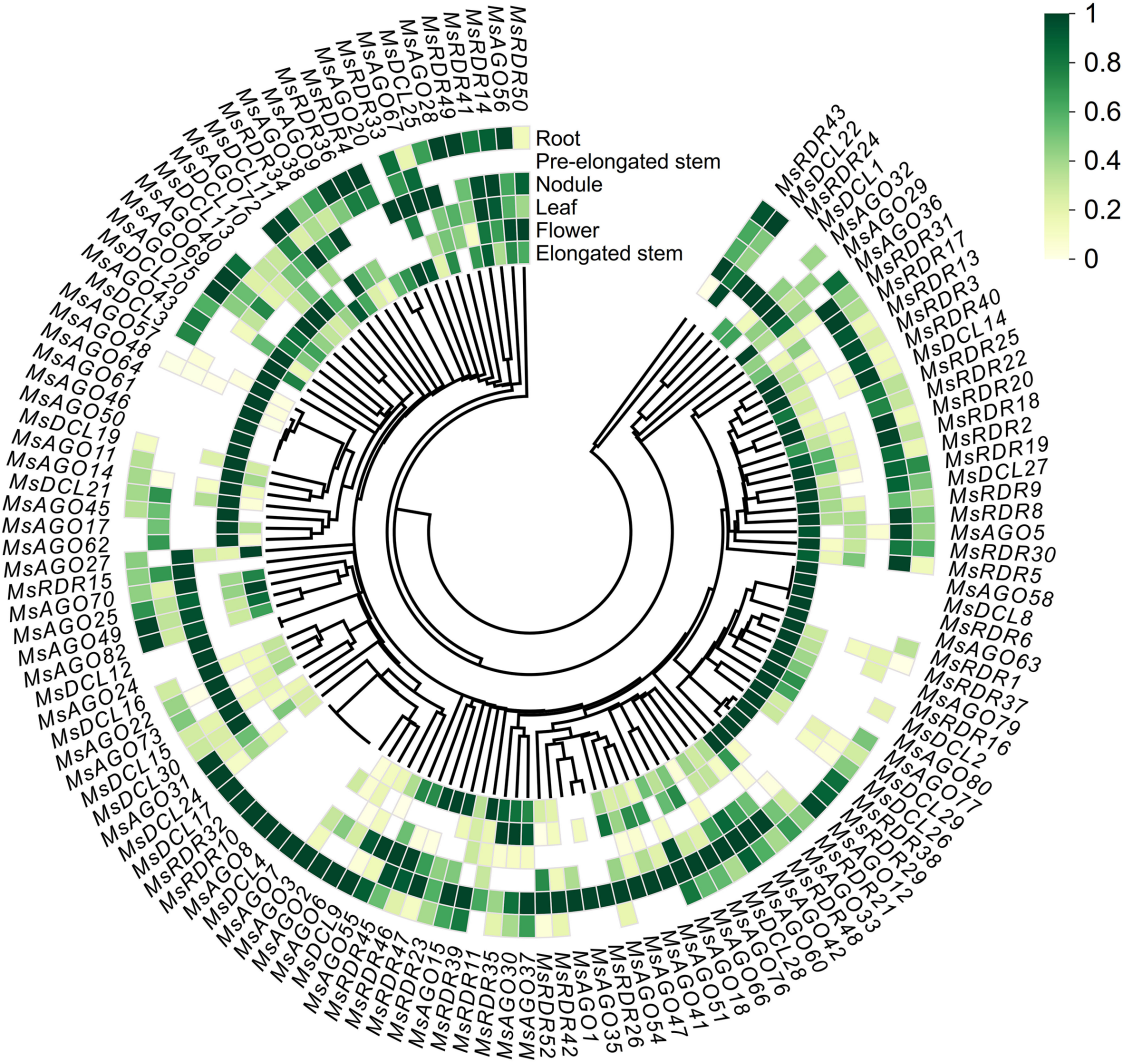
Relative expression levels of RNA silencing pathway genes across six alfalfa tissues. The circular heatmap displays the normalized expression profiles of *MsDCL*, *MsAGO*, and *MsRDR* genes in roots, pre-elongated stems, nodules, leaves, flowers, and elongated stems, arranged from the outermost to the innermost ring. Color intensity ranges from yellow to dark green, indicating increasing gene expression levels. Blank (uncolored) cells represent undetectable expression. Expression data were normalized using the MinMaxScaler method (row-wise) and visualized via the Chiplot platform. Hierarchical clustering was performed using the single linkage method.

To investigate the tissue-specific expression patterns of RNA silencing pathway genes in alfalfa, RNA-seq data from six distinct tissues—roots, leaves, flowers, elongated stems, pre-elongated stems, and nodules—were analyzed. Among the 165 identified RNA silencing pathway genes, 133 genes exhibited detectable expression in at least one of these tissues. The remaining 32 genes, including six *MsDCL* genes (*MsDCL5*, *MsDCL6*, *MsDCL7*, *MsDCL18*, *MsDCL23*, *MsDCL31*), 20 *MsAGO* genes (*MsAGO4*, *MsAGO10*, *MsAGO13*, *MsAGO16*, *MsAGO19*, *MsAGO21*, *MsAGO23*, *MsAGO26*, *MsAGO34*, *MsAGO39*, *MsAGO44*, *MsAGO52*, *MsAGO53*, *MsAGO59*, *MsAGO65*, *MsAGO68*, *MsAGO71*, *MsAGO74*, *MsAGO78*, *MsAGO81*), and six *MsRDR* genes (*MsRDR7*, *MsRDR12*, *MsRDR27*, *MsRDR28*, *MsRDR44*, *MsRDR51*), showed no detectable expression, implying that these genes may function in other tissues or in response to specific biotic or abiotic stresses. Among the 133 expressed genes, several displayed strong tissue specificity. Notably, *MsAGO46*, *MsAGO50*, and *MsAGO61* were exclusively expressed in flowers; *MsRDR24* was detected only in leaves; *MsDCL12* and *MsAGO24* were specifically expressed in nodules; and *MsRDR26* was confined to pre-elongated stems. Moreover, six genes—*MsDCL4*, *MsDCL17*, *MsAGO7*, *MsAGO8*, *MsRDR10*, and *MsRDR32*—were uniquely expressed in roots (**Figure 6**; **Supplementary Table S8**). While many genes exhibited expression across multiple tissues, their expression levels varied considerably, indicating potential functional divergence and tissue-specific regulatory mechanisms.

### 3.8 RT-qPCR analysis of RNA silencing pathway genes expression patterns under abiotic stress

**Figure 7:**
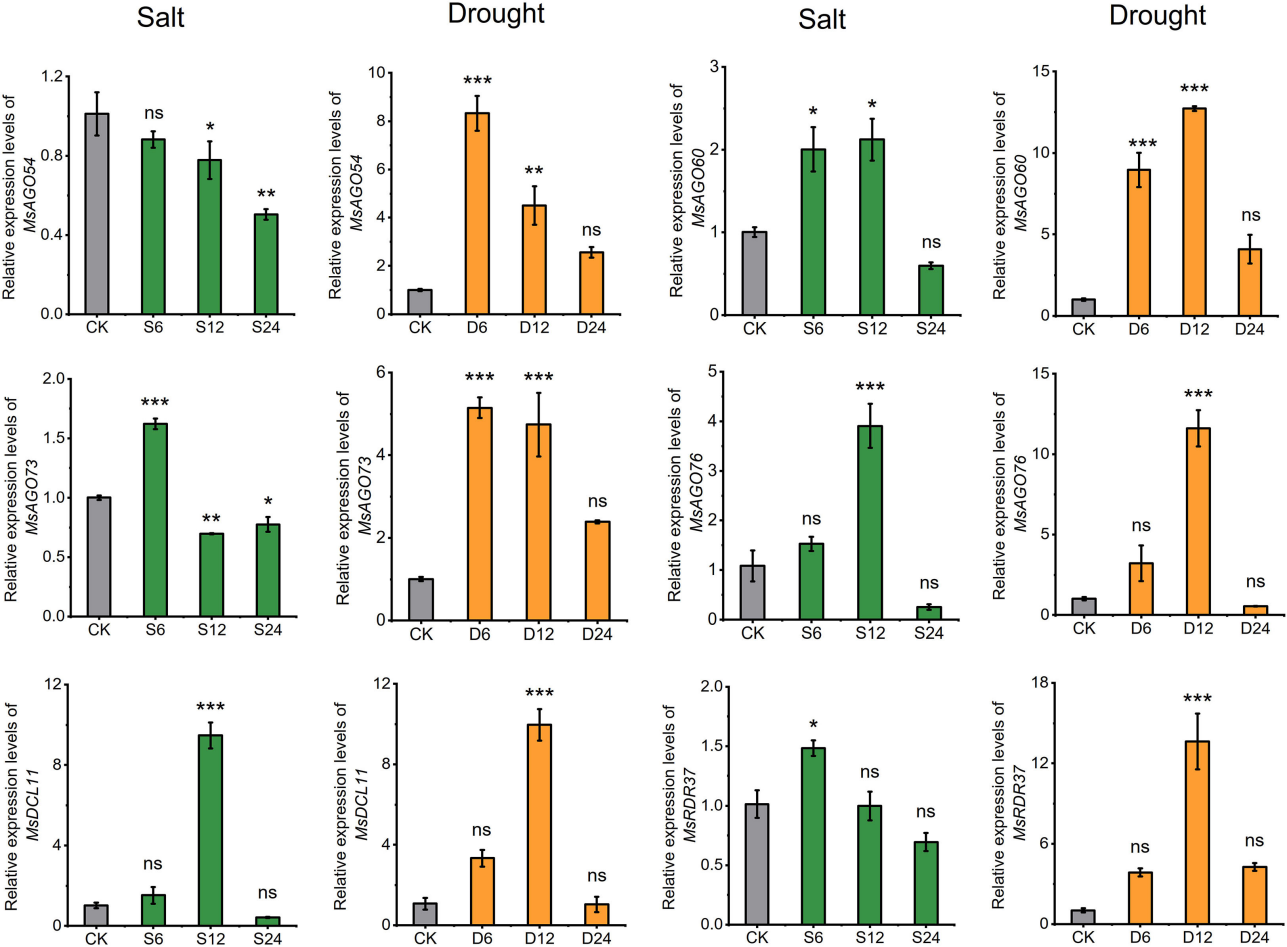
Expression profiles of six selected *MsDCL*, *MsAGO*, and *MsRDR* genes under salt and drought stress conditions, as determined by RT-qPCR. Green bars indicate relative expression levels under salt stress, while orange bars indicate those under drought stress. S6, S12, and S24 represent samples collected at 6, 12, and 24 hours after salt treatment, respectively. D6, D12, and D24 denote the corresponding time points under drought stress. The expression level at 0 hours was used as the control (CK). Statistical significance was assessed using Student’ s *t*-test. *, *P* < 0.05; **, *P* < 0.01; ***, *P* < 0.001; ns, not significant.

To confirm the consistency of expression patterns with RNA-seq data for genes in the RNA silencing pathway under abiotic stress, six genes (*MsDCL11*, *MsAGO54*, *MsAGO60*, *MsAGO73*, *MsAGO76*, and *MsRDR37*) were selected based on their expression changes under salt and drought conditions and verified by RT-qPCR under corresponding treatments (**Supplementary Figure S5**; **Figure 7**). *MsDCL11*, *MsAGO60*, and *MsAGO76* were significantly upregulated at 6 h and 12 h, followed by downregulation at 24 h. *MsAGO73* and *MsRDR37* showed a similar trend but responded more rapidly, with their expression beginning to decline as early as 12 h. In contrast, the expression of *MsAGO54* decreased continuously and progressively throughout the 24 h salt treatment. Under drought stress, all six genes displayed a similar expression pattern, characterized by an initial significant upregulation followed by a gradual decline. Among them, *MsAGO54* and *MsAGO73* responded more rapidly, with their expression starting to decrease by 12 h, while the other genes maintained an upward trend within the first 12 h. Notably, the expression pattern of *MsAGO76* and *MsDCL11* was consistent under both salt and drought conditions.

## 4 Discussion

RNA silencing is a conserved regulatory mechanism mediated by small RNAs, which plays essential roles in plant development and stress responses (Zhao and Guo, 2022). In model and crop species such as *O. sativa* (Kapoor et al., 2008), *G. max* (Liu et al., 2014), and *T. aestivum* (Mishra et al., 2023), three core gene families—Dicer-like (*DCL*), Argonaute (*AGO*), and RNA-dependent RNA polymerase (*RDR*)—have been systematically characterized. In this study, we comprehensively identified 31 *MsDCL*, 82 *MsAGO*, and 52 *MsRDR* genes in autotetraploid alfalfa based on the high-quality Xinjiang Daye genome assembly. Furthermore, the phased autotetraploid genome assembly of Xinjiang Daye enabled us to discern homeologous gene copies and assess their retention patterns. In many cases, we observed a clear 1:4 relationship among homeologous loci for *MsDCL*, *MsAGO*, and *MsRDR* genes, consistent with the tetraploid nature of alfalfa. However, not all homeologs were retained equally across subgenomes, suggesting potential selective loss or functional divergence following polyploidization. Such uneven retention may reflect subgenome-specific evolutionary pressures or expression biases, which could influence stress-responsive regulatory networks. These observations underscore the importance of using phased, allele-aware genomes in polyploid species to accurately capture gene family diversity and to elucidate the evolutionary trajectories of duplicated genes in adaptive processes (Yang et al., 2025). The uneven chromosomal distribution of *DCL*, *AGO*, and *RDR* genes, with some chromosomes lacking these family members (**Figure 1**), may be attributed to several factors, including subgenome-specific gene loss in this autotetraploid, chromosomal rearrangements during evolution, or potential limitations in genome annotation. Future studies will help clarify their distribution patterns.

Compared with other species, alfalfa harbors a larger repertoire of RNA silencing pathway genes. This expansion may be attributed to two main factors. First, compared to haploid genomes, the identification of gene family members in tetraploid genomes is more comprehensive. This approach prevents gene loss during chromosome reduction and more accurately reflects the ancestral size and diversity of gene families (Panchy et al., 2016). A trend also observed in cotton, where the tetraploid cotton genome has approximately twice as many *MIOX* genes as the diploid cotton genome (Li et al., 2021b). Second, frequent tandem and segmental duplications have likely driven further expansion. For example, rice contains only three tandem duplication events (*OsAGO4a/15*, *OsAGO2/3*, and *OsAGO11/12*), and three pairs of segmental duplication events (*OsAGO1a/1b*, *OsAGO13/14*, and *OsDCL2a/2b*) (Kapoor et al., 2008), while alfalfa exhibits markedly more, particularly for *MsAGO* and *MsRDR* genes (**Figures 3**, **4**). Consequently, the higher frequency of such events in alfalfa may have facilitated the functional divergence of RNA silencing pathway genes.

Gene duplication, including both tandem and segmental events, is a major force driving functional innovation (Moore and Purugganan, 2003; Cannon et al., 2004; Magadum et al., 2013). We observed extensive tandem duplication of *MsRDR* genes on chromosomes chr1.1–chr1.4, a pattern not previously reported in other species. This lineage-specific expansion may result from the unique genome structure or evolutionary trajectory of *M. sativa*, though further comparative and functional genomic analyses are needed to elucidate its biological significance (Leister, 2004). Interestingly, tandemly arrayed MsRDR1–11 were classified into two separate events due to their low sequence identity (e.g., *MsRDR1 vs*. *MsRDR2*: 32.8%), implying ancient divergence and potential functional differentiation. Overall, segmental duplication was the dominant mechanism underlying RNA silencing gene family expansion in alfalfa. The occurrence of motif/domain loss and extremely low or undetectable expression in some gene copies may indicate functional divergence or loss of function in these genes, while the definitive classification of such genes requires further transcript and functional validation experiments.

Phylogenetic analysis classified *MsDCL* genes into four major clades, each corresponding to one *A. thaliana* DCL ortholog, consistent with reports in *Eremochloa ophiuroides* (Liu et al., 2024), *Citrus sinensis* (Mosharaf et al., 2020), and *Capsicum annuum* (Qin et al., 2018). Notably, *MsDCL19–*22 clustered with *AtDCL1* and exhibited flower-specific expression (**Figure 6**), suggesting their involvement in floral development, similar to *AtDCL1*’s known role in promoting flowering (Schmitz et al., 2007). The *AGO* gene family in angiosperms generally falls into three clades: *AGO1/5/10*, *AGO2/3/7*, and *AGO4/6/8/9* (Zhang et al., 2015), a classification recapitulated in our analysis. Notably, *MsAGO66–*76 in the *AGO1* clade contain the characteristic Gly-rich_Ago1 domain and share close homology with *AtAGO1*, which is central to miRNA-mediated silencing (Bologna and Voinnet, 2014; Mishra et al., 2023). Furthermore, all but one gene (*MsAGO21*) in the *AGO4/6/8/9* clade are predicted to localize in the nucleus, supporting their roles in transcriptional silencing pathways, whereas *MsAGO21* may have diverged in function due to its distinct subcellular localization. The *MsRDR* gene family was also divided into four subgroups, with subgroup I being the largest due to extensive tandem duplications. This group showed high homology to *AtRDR1*, which mediates antiviral defense by producing virus-derived siRNAs (Cao et al., 2014). Functional analogs such as *NtRDR1* and *SlRDR1a* have been shown to suppress viroid replication and enhance viral resistance (Li et al., 2021a), suggesting that *MsRDR* subgroup I genes may serve similar roles in early antiviral responses in alfalfa.

To further investigate their potential stress-responsive functions, we conducted analysis of *cis*-elements in the promoters of genes involved in the RNA silencing pathway. Predictive analysis revealed that most *MsDCL*, *MsAGO*, and *MsRDR* genes contain hormone- and stress-related elements, with those associated with abscisic acid, anaerobic induction, and methyl jasmonate (MeJA) signaling being particularly prominent (**Figure 5**; **Supplementary Table S7**). These in silico predictions are consistent with findings reported in other herbaceous plants (Liu et al., 2024). However, the functional implications of these cis-elements require further experimental validation. Transcriptomic analysis under salt and drought stress conditions revealed six stress-responsive genes: *MsAGO54*, *MsAGO60*, *MsAGO73*, *MsAGO76*, *MsDCL11*, and *MsRDR37* (**Figure 7**). Among these, *MsAGO73* and *MsAGO76*—both closely related to *AtAGO1* with >80% sequence identity—exhibited a dynamic expression pattern: an initial increase followed by a subsequent decrease in their expression levels under salt stress. This is consistent with studies showing that *AtAGO1* modulates stress-responsive miRNA networks and is upregulated under salt conditions (Várallyay et al., 2010; Li et al., 2012; Dolata et al., 2016). Existing studies have demonstrated that drought stress enhances the transcriptional activity of *AGO1*, while moderately reducing its functional level can improve plant drought tolerance (Li et al., 2012). In conjunction with the RT-qPCR results from this study, two *MsAGO* gene family members (*MsAGO73* and *MsAGO76*) exhibiting high homology to *AGO1* were upregulated during the early stage of drought stress, which may represent a direct response to drought conditions. The subsequent downregulation in their expression at later stages suggests that this dynamic modulation may serve as an important mechanism for alfalfa to cope with prolonged drought stress. Analysis of RT-qPCR results for other genes indicates that *MsAGO54*, *MsAGO60*, *MsDCL11*, and *MsRDR37* may employ a similar regulatory strategy in response to drought stress. Notably, the drought response mechanism of *MsRDR37* is potentially associated with drought-responsive elements present in its promoter region, which may participate in drought adaptation by modulating gene expression.

In summary, the expansion, structural features, and stress-responsive expression patterns of *MsDCL*, *MsAGO*, and *MsRDR* genes underscore their crucial roles in RNA silencing and adaptive responses. Compared to diploid self-pollinating species, the use of a high-quality phased genome for autotetraploid alfalfa enables more accurate gene identification—including allelic resolution and the detection of subgenome-specific gene retention or expression bias. This approach offers deeper insights into the evolutionary diversification and regulatory specialization of RNA silencing genes in this highly heterozygous, outcrossing species. Furthermore, the functional delineation of key family members within the small RNA pathway has been preliminarily clarified: *DCL1*–*AGO1* typically mediates miRNA-based post-transcriptional silencing, RDR6 together with *DCL4/DCL2* generates secondary siRNAs, while *RDR2–DCL3* is primarily involved in producing 24-nt siRNAs and facilitating the RdDM pathway (Vaucheret, 2006). These mechanistic insights establish a critical foundation for future systematic investigations into how *AGO*, *DCL*, and *RDR* family members regulate miRNAs or siRNAs to enhance alfalfa’s tolerance to abiotic stress, thereby supporting subsequent functional studies on their roles in stress resilience and regulatory network integration.

## 5 Conclusion

In this study, we systematically identified 31 *MsDCL*, 82 *MsAGO*, and 52 *MsRDR* genes in the *M. sativa* genome. Phylogenetic analyses grouped these genes into four (*MsDCL*), three (*MsAGO*), and four (*MsRDR*) subfamilies, respectively, with members within each subgroup exhibiting conserved gene structures and motif compositions. Both segmental and tandem duplications were found to play significant roles in the expansion and diversification of these gene families. Promoter analysis revealed the presence of multiple *cis*-acting elements related to hormone signaling and abiotic stress responsiveness. By integrating RNA-seq datasets from salt and drought stress conditions, we identified six key stress-responsive genes—*MsAGO54*, *MsAGO60*, *MsAGO73*, *MsAGO76*, *MsDCL11*, and *MsRDR37*—and validated their differential expression patterns under stress using RT-qPCR. The distinct expression profiles suggest that these RNA silencing pathway genes may play important regulatory roles in alfalfa’s adaptive responses to abiotic stress. Together, our findings provide a comprehensive foundation for future functional studies of RNA silencing components in alfalfa and contribute to the broader understanding of their roles in stress resilience and gene regulation.

## Data Availability

The original contributions presented in the study are included in the article/**Supplementary Material**. Further inquiries can be directed to the corresponding authors.

## Supplementary material

Supplementary Figure 1 Basic information of RNA silencing pathway protein motifs: Each protein family contains 10 conserved motifs, with each motif characterized by four fundamental parameters: E-value, sites, width, and sequence.

Supplementary Figure 2 Gene structure and conserved motif analysis of *MsDCL* and *MsRDR* families. **(A)** Phylogenetic tree of both gene families. **(B)** Conserved motifs identified in the two gene families. **(C)** Gene structures and functional domains of both families.

Supplementary Figure 3 Gene structure and conserved motif analysis of *MsAGO* gene family. **(A)** Phylogenetic tree of *MsAGO* gene family. **(B)** Conserved motifs identified in *MsAGO* gene family. **(C)** Gene structures and functional domains of *MsAGO* gene family.

Supplementary Figure 4 Schematic diagram of the syntenic relationships of *MsDCL* and *MsRDR* genes in alfalfa. The outermost colored circles represent the 32 chromosomes of alfalfa, and the position of *MsDCL* and *MsRDR* genes on the chromosome is marked on the circle. The gray ribbons represent syntenic blocks in the alfalfa genome, and the segmental duplication events are marked in red.

Supplementary Figure 5 Expression of RNA silencing genes in alfalfa under stress. **(A)** Expression of RNA silencing genes in alfalfa under drought stress. Under drought stress: 0 h as CK and 1, 3, 6, 12 and 24 h as D1, D2, D3, D4 and D5, respectively. **(B)** Expression of RNA silencing genes in alfalfa under salt stress. Under salt stress: 0 h as CK and 0.5, 1, 3, 6, 12 and 24 h as S1 to S6, respectively. **(C)** Venn diagram of RNA silencing pathway genes responding to drought and salt stresses in alfalfa.
