# Supplementary material for: Genome-wide identification and characterization of DCL, AGO, and RDR gene families and their abiotic stress responses in alfalfa (Medicago sativa L.)
Source: Front Plant Sci. 2026 Feb 16;17:1753305. doi: 10.3389/fpls.2026.1753305 (PMC12950779; doi:10.3389/fpls.2026.1753305)
Supplement: Supplementary Figure 1 — Basic information of RNA silencing pathway protein motifs: Each protein family contains 10 conserved motifs, with each motif characterized by four fundamental parameters: E-value, sites, width, and sequence. [file DataSheet1.pdf]

DCL		E-value	Sites	Width	Sequence
Motif1	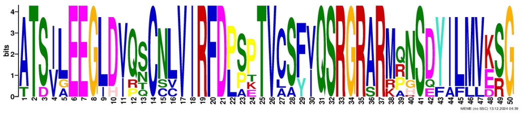	2.4×10^{-1248}	31	50	ATSILEEGLDVQSCNLVIRFDPSPTVCSFVQSRGRARMQNSDYILMVKSG
Motif2	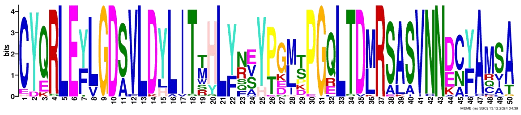	5.8×10^{-1179}	31	50	CYQRLEYLGDSVLDYLITTHLYNEYPGMTPGQLTDMRSASVNNDCYAMSA
Motif3	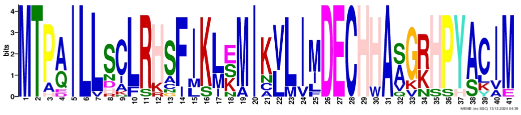	1.8×10^{-928}	30	41	MTPAILLSCLRHSFIKLEMIKVLIMDECHHASGRHPYACIM
Motif4	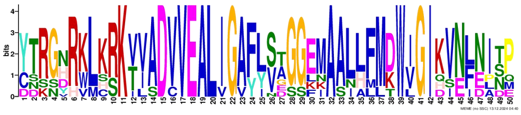	7.3×10^{-1073}	31	50	YTRGNRKLKRKVVADVVEALIGAFLSTGGEMAALLFMDWIGIKVNLNITP
Motif5	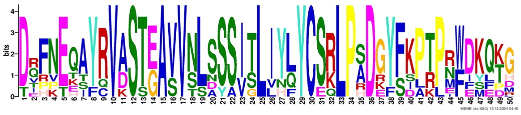	5.8×10^{-1045}	31	50	DRFNEQAYRVASTEAVVNLSSSITLIYLYCSRLPSDGYFKPTPRWDKQKG
Motif6	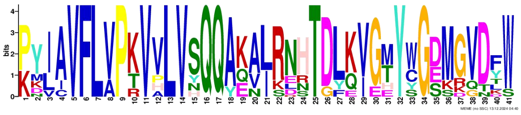	1.4×10^{-888}	30	41	PYIAVFLVPKVVLVSQQAKAJRNHTDLKVGMYWGDMGVDFW
Motif7	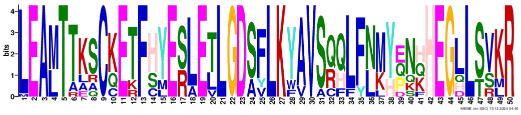	4.7×10^{-1081}	29	50	LEAMTTKSCKETFHYESLETLGDSFLKYAVSQQLFNMYENHHEGLLSVKR
Motif8	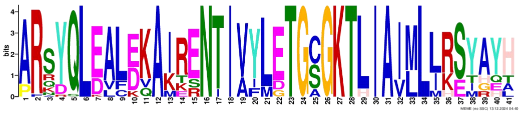	1.4×10^{-863}	30	41	ARSYQLEALEKAIRENTIVYLETGCGKTLIAIMLLRSYAYH
Motif9	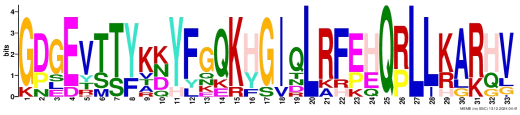	1.5×10^{-648}	31	33	GDGEVTTYKKYFGQKHGIQLRFEHQRLLKARHV
Motif10	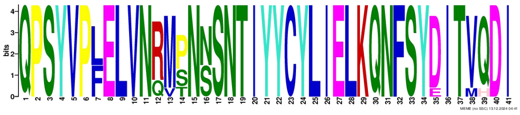	1.1×10^{-641}	18	41	QPSYVPLELVNRMPNNSNTIYYCYLIELKQNFSYDITVQDI

AGO		E-value	Sites	Width	Sequence
Motif1	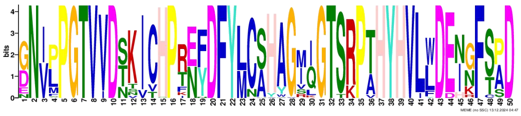	1.3×10^{-3708}	79	50	GNIPPGTVVDSKICHPREFDFYLCSHAGMIGTSRPTHYHVLWDENGFSPD
Motif2	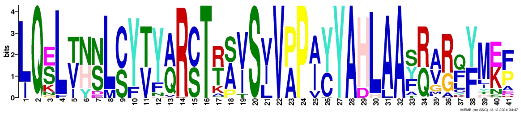	1.5×10^{-2479}	77	41	LQELTNNLCYTYARCTRAVSVVPPAYYAHLAASRARQYMEF
Motif3	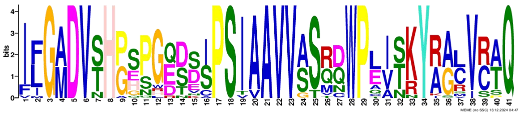	5.0×10^{-2493}	77	41	IFGADVSHPSPGZDSIPSIAAVVASRDWPLISKYRALVRAQ
Motif4	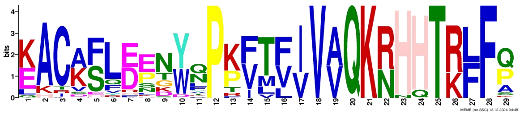	2.1×10^{-1685}	80	29	KACAFLEENYQPKFTFIVAQKRHHTRLFQ
Motif5	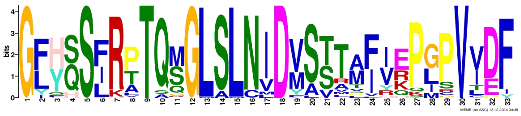	3.6×10^{-1901}	80	33	GFHSSFRPTQMGLSLNIDVSTTAFIEPGPVVDF

Motif6	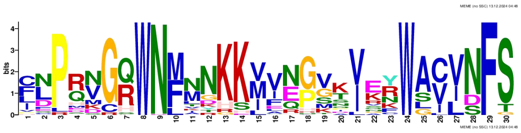	6.6×10^{-1395}	75	30	CNPRNGQWNMNNKKVVNGVKVERWACVNFS
Motif7	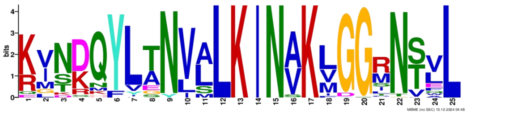	8.5×10^{-1359}	80	25	KVNDQYLTNVALKINAKLGGRNSVL
Motif8	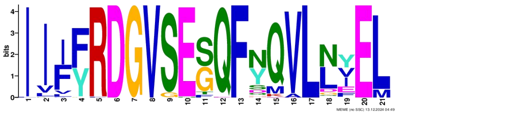	3.7×10^{-1278}	78	21	IIIFRDGVSESQFNQVLNYEL
Motif9	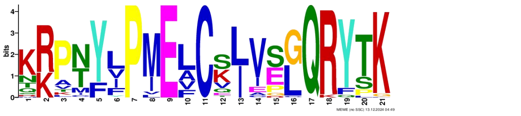	5.0×10^{-1133}	80	21	KRPNYLPMELCSJVSGQRYTK
Motif10	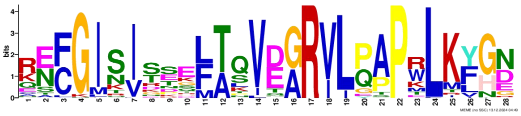	1.5×10^{-1341}	82	28	REFGISISSELTQVEGRVLPAPRLKYGB
**RDR**		E-value	Sites	Width	Sequence
Motif1	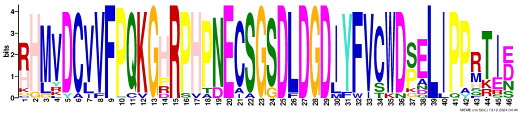	1.7×10^{-2114}	51	46	RHMVDCVVFPQKGHRPHPNECSGSDLDGDIYFVCWDSELIPPRTIE
Motif2	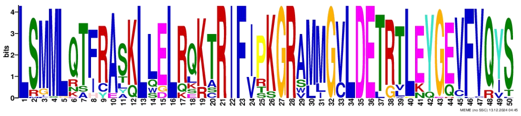	7.2×10^{-2031}	48	50	LSMMLQTFRASKLLELRQKTRIFIPKGRAMMGVLDETRTLEYGEVFVQYS
Motif3	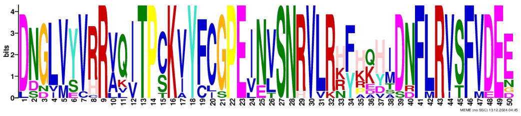	4.7×10^{-2000}	48	50	DNGLVYVRRVQITPCKVYFCGPEINVSNRVLRHFHQHIDNFLRVSFVDEE
Motif4	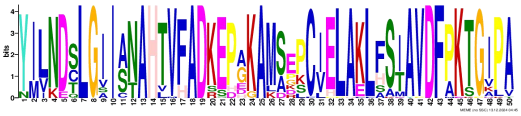	8.2×10^{-1988}	48	50	YILNDSLGIIANAHTVFADKEPAKAMSEPCIELAKLFSIAVDFPKTGIPA
Motif5	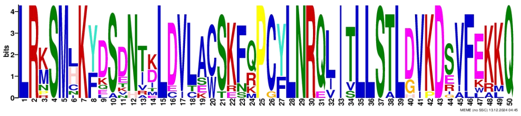	7.3×10^{-1931}	48	50	LRKSMLKYDSDNTKLDVLACSKFQPCYLNRQLITLLSTLDVKDSVFEKKQ
Motif6	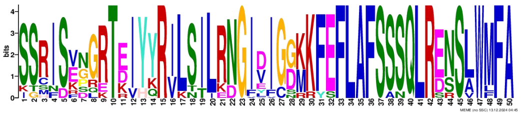	8.2×10^{-1941}	48	50	SSRISVNGRTEIYYRILSILRNGIDIGGKKFEFLAFSSSQLRENSLWMFA
Motif7	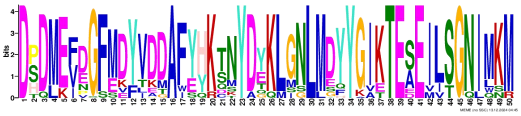	3.9×10^{-1906}	48	50	DPDMEFDGFMDYVDDAFYHKTNYDYKLGNLMDYYGIKTESEILSGNIMKM
Motif8	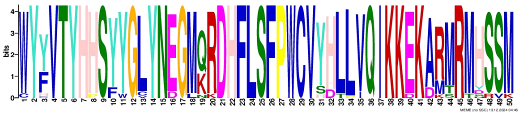	4.1×10^{-1811}	36	50	WYYVTYHHSYYGLYNEGMQRDHFLSFPWCVYHLLVQIKKEKARMRMHSSM
Motif9	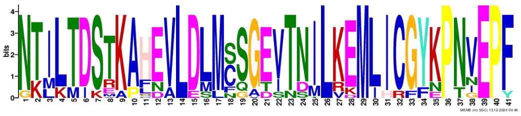	3.9×10^{-1417}	43	41	NTILTDSTKAHEVLDLMSSGEVTNILKEMLICGYKPNVEPF
Motif10	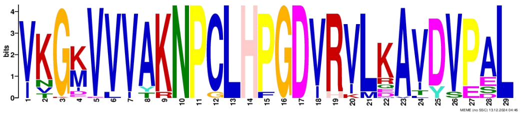	1.0×10^{-1238}	52	29	VKGKVVVAKNPCLHPGDVRVLK
