# Supplementary figures and images for: Genome-wide identification and characterization of DCL, AGO, and RDR gene families and their abiotic stress responses in alfalfa (Medicago sativa L.)
Source: Front Plant Sci. 2026 Feb 16;17:1753305. doi: 10.3389/fpls.2026.1753305 (PMC12950779; doi:10.3389/fpls.2026.1753305)

A

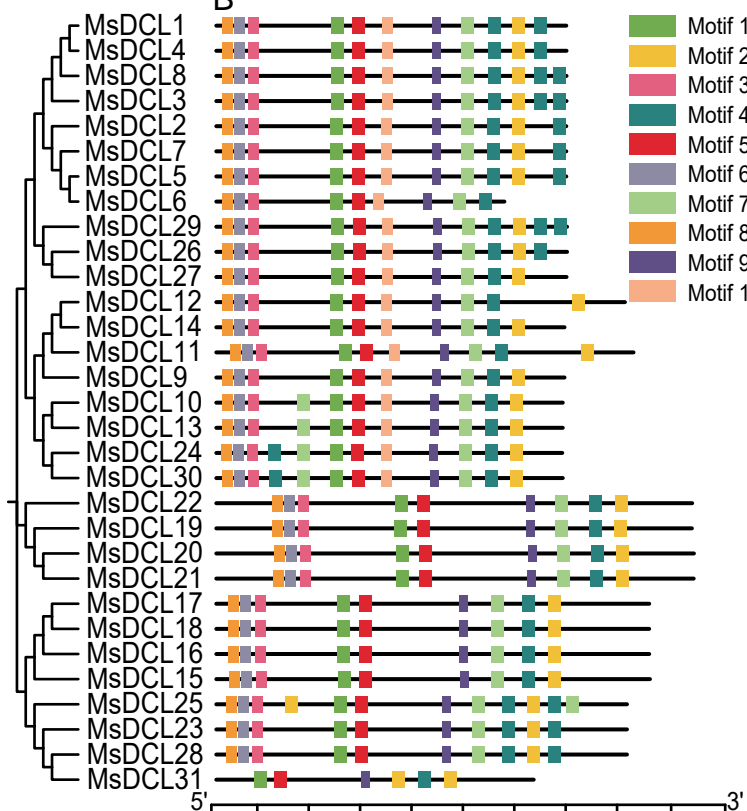

B

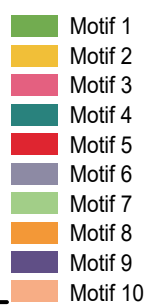

C

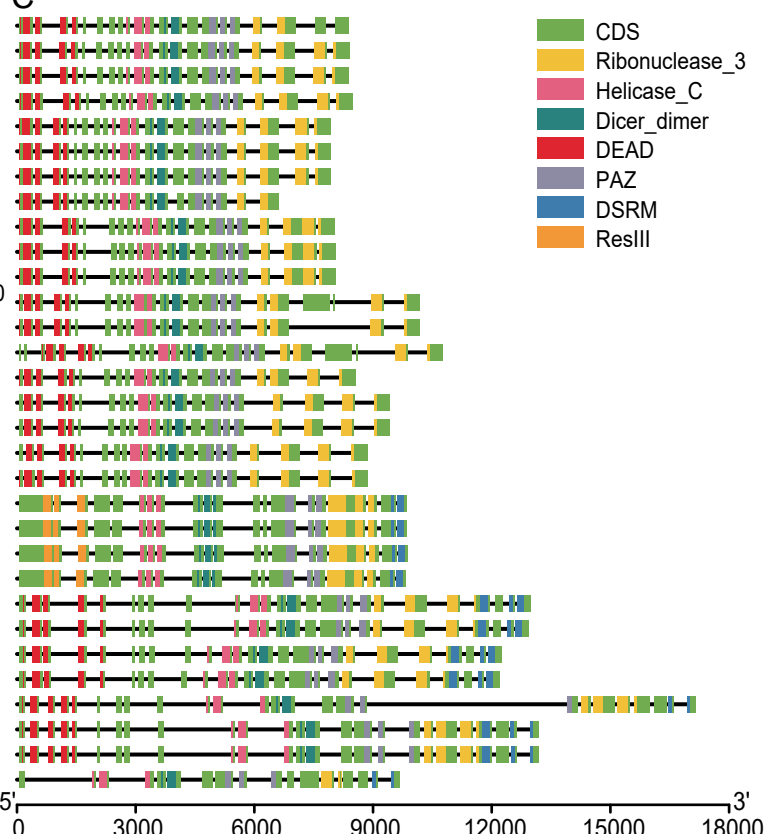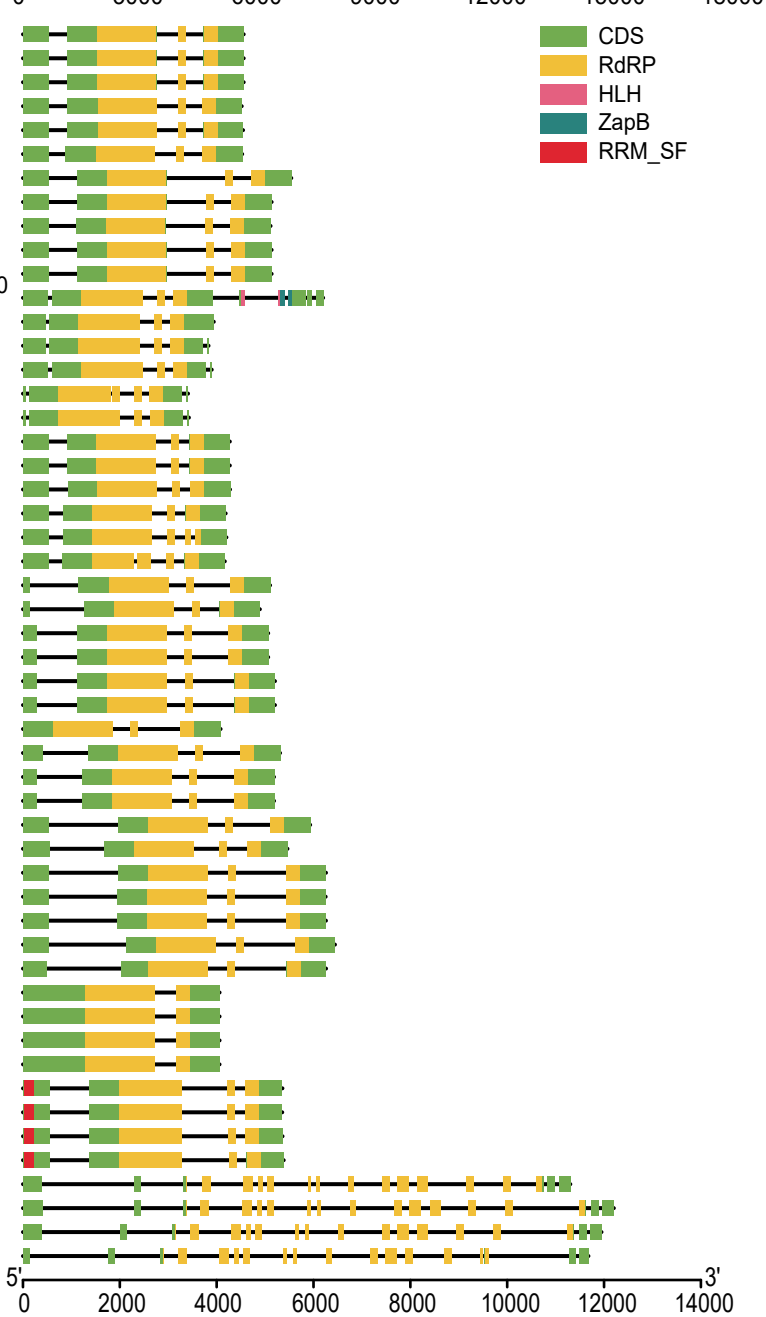

Supplement: Supplementary Figure 2 — Gene structure and conserved motif analysis of MsDCL and MsRDR families. (A) Phylogenetic tree of both gene families. (B) Conserved motifs identified in the two gene families. (C) Gene structures and functional domains of both families. [file DataSheet2.pdf]

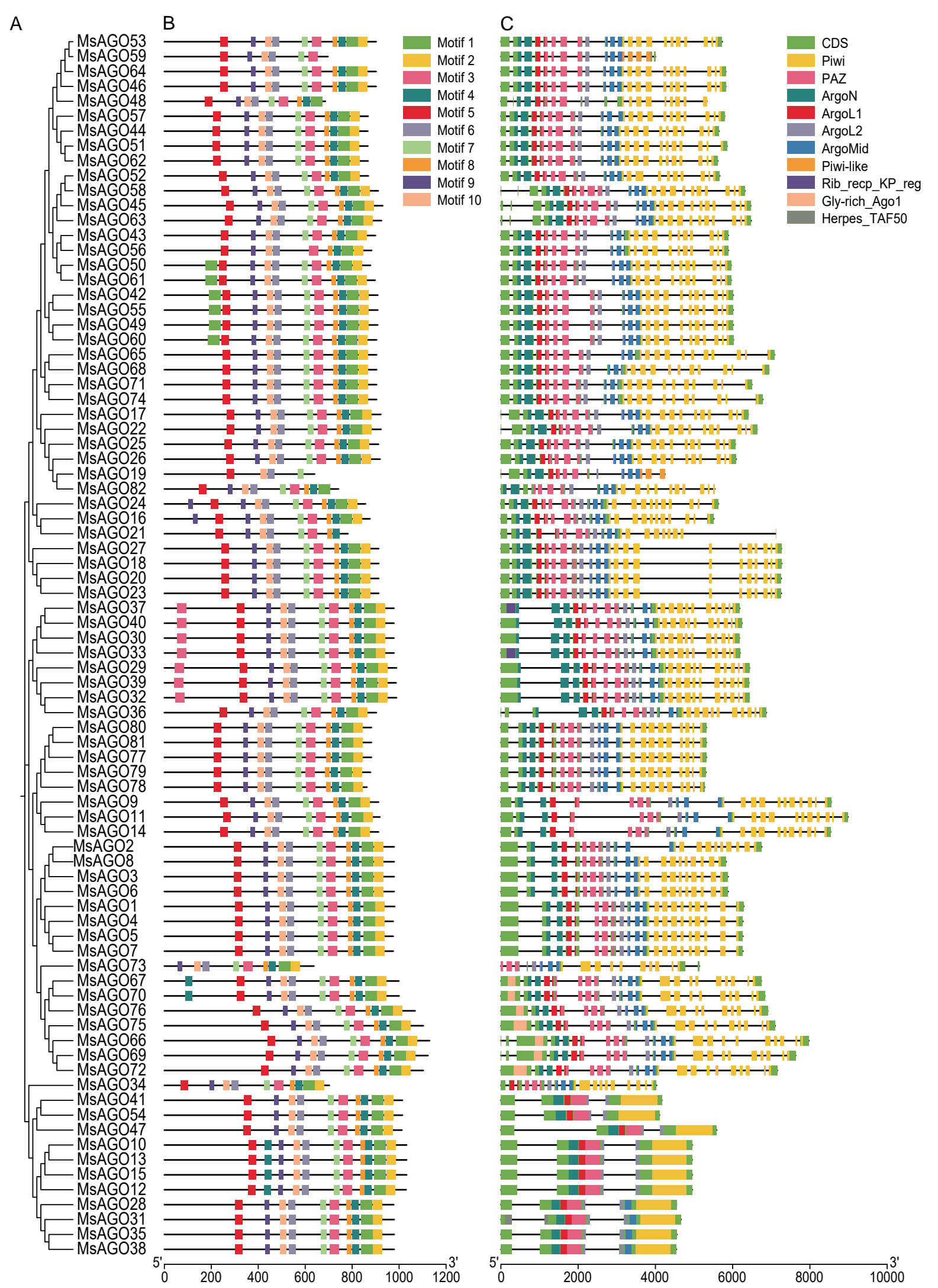

Supplement: Supplementary Figure 3 — Gene structure and conserved motif analysis of MsAGO gene family. (A) Phylogenetic tree of MsAGO gene family. (B) Conserved motifs identified in MsAGO gene family. (C) Gene structures and functional domains of MsAGO gene family. [file DataSheet3.pdf]

A

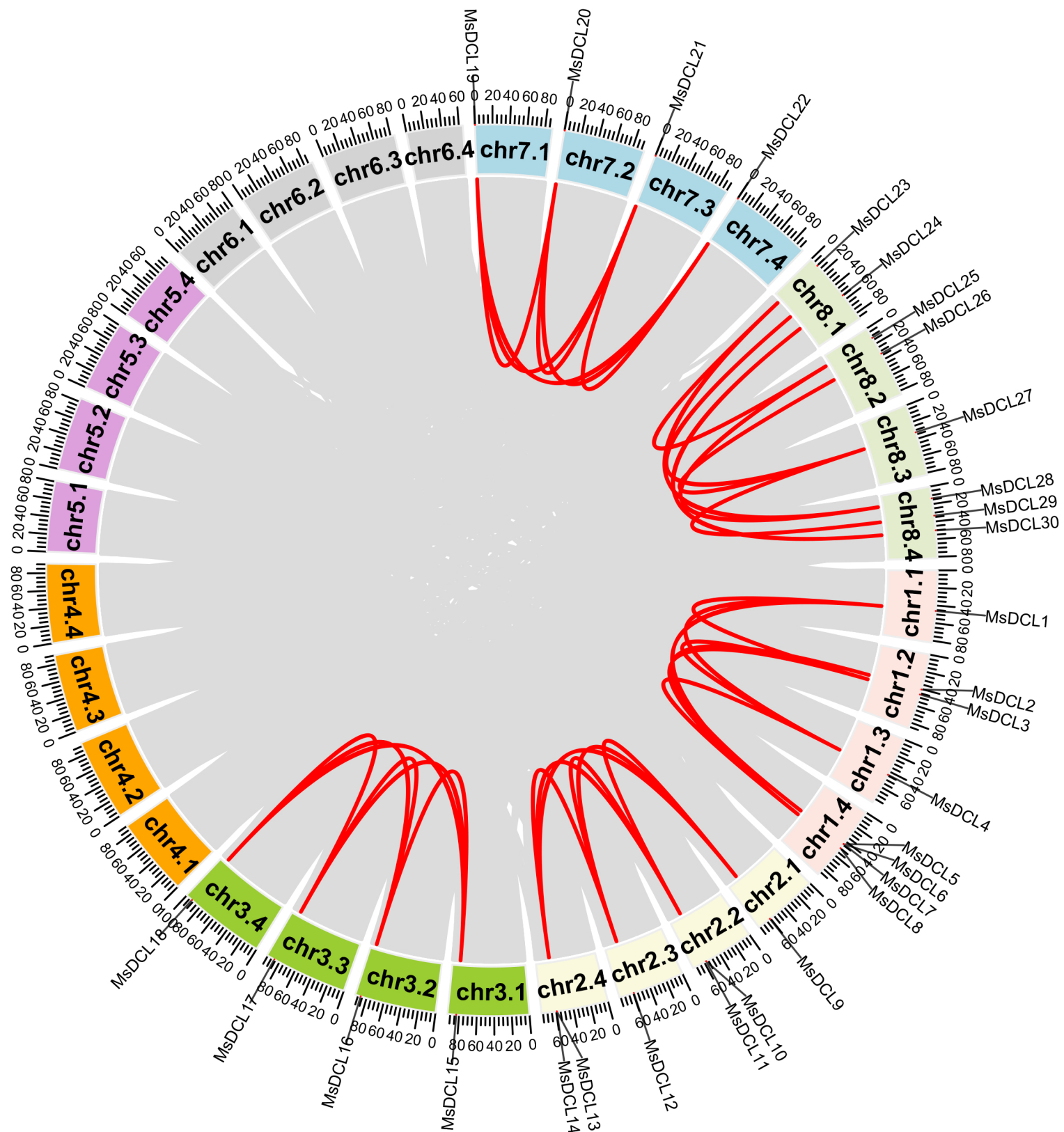

B

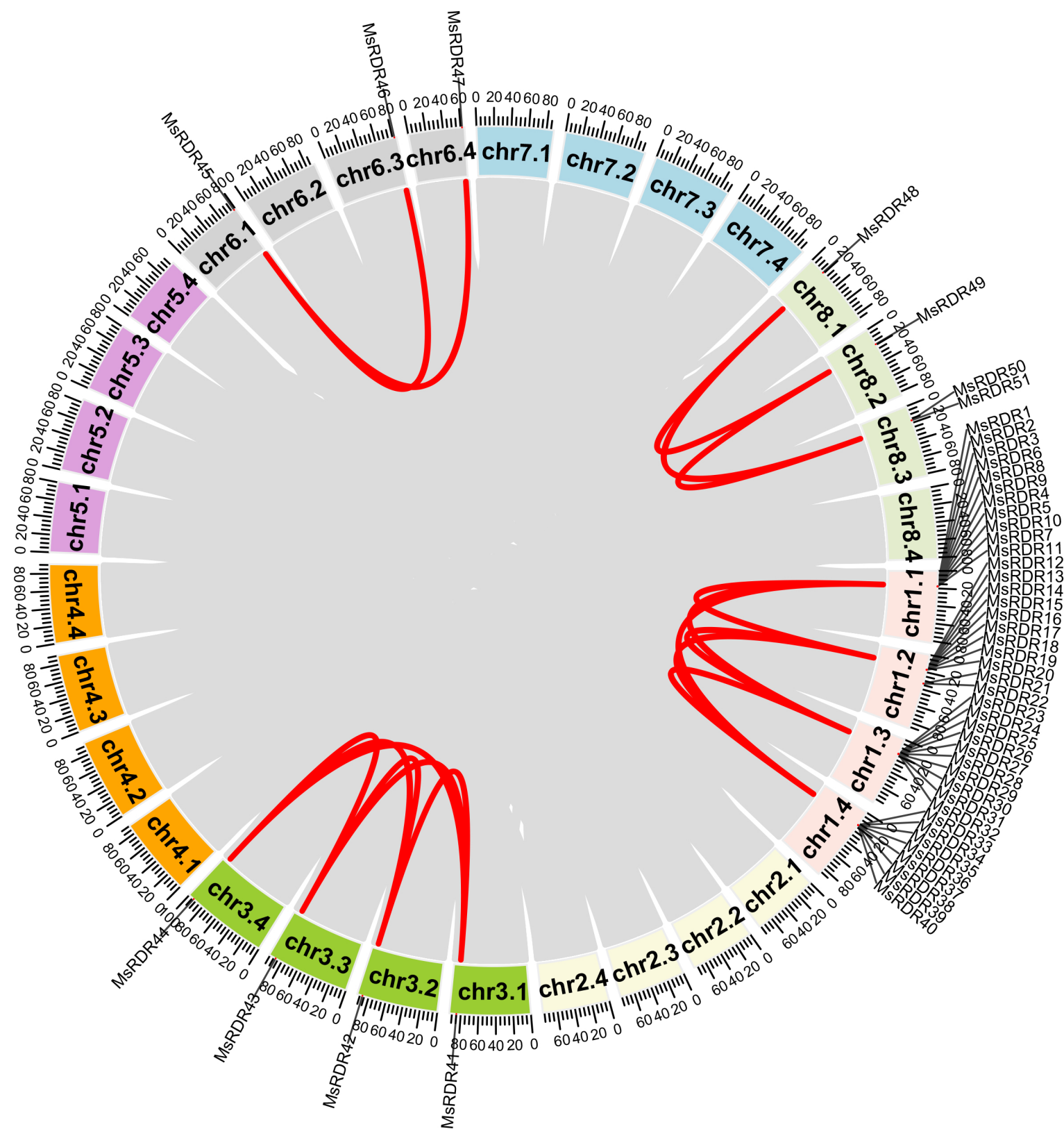

Supplement: Supplementary Figure 4 — Schematic diagram of the syntenic relationships of MsDCL and MsRDR genes in alfalfa. The outermost colored circles represent the 32 chromosomes of alfalfa, and the position of MsDCL and MsRDR genes on the chromosome is marked on the circle. The gray ribbons represent syntenic blocks in the alfalfa genome, and the segmental duplication events are marked in red. [file DataSheet4.pdf]

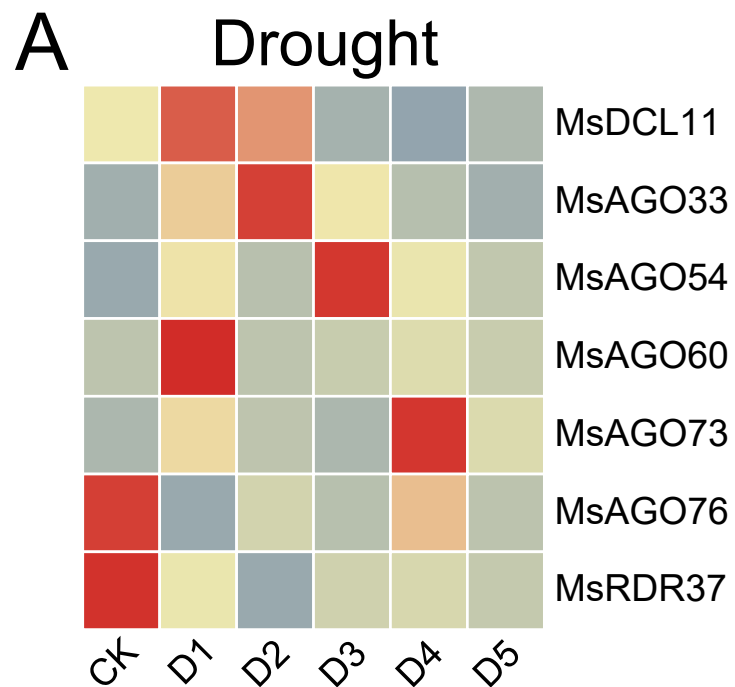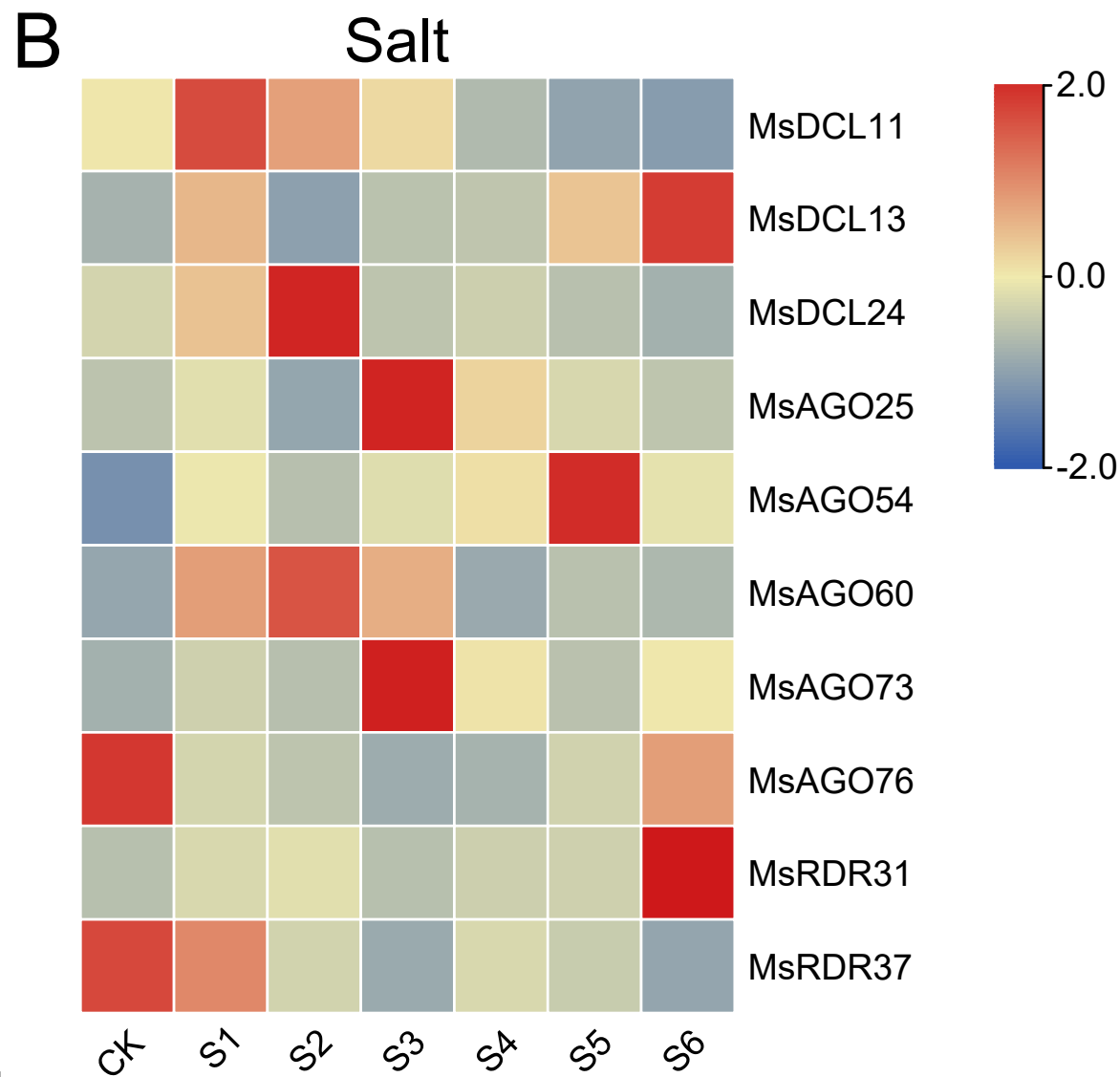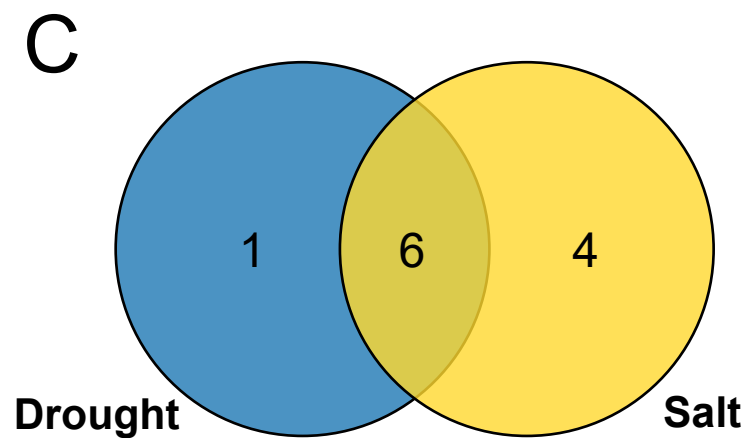

Supplement: Supplementary Figure 5 — Expression of RNA silencing genes in alfalfa under stress. (A) Expression of RNA silencing genes in alfalfa under drought stress. Under drought stress: 0 h as CK and 1, 3, 6, 12 and 24 h as D1, D2, D3, D4 and D5, respectively. (B) Expression of RNA silencing genes in alfalfa under salt stress. Under salt stress: 0 h as CK and 0.5, 1, 3, 6, 12 and 24 h as S1 to S6, respectively. (C) Venn diagram of RNA silencing pathway genes responding to drought and salt stresses in alfalfa. [file DataSheet5.pdf]
